# Computational study of the relative stability of some glass-ionomer cement-forming molecules

**DOI:** 10.1007/s00894-022-05211-x

**Published:** 2022-09-28

**Authors:** Jair Gaviria, Silvia Quijano, Jairo Quijano, Pablo Ruiz

**Affiliations:** 1 Laboratorio de Fisicoquímica Orgánica, Facultad de Ciencias, Universidad Nacional de Colombia, 050034 Medellin, Colombia; 2 Programa de Microbiología, Facultad de Ciencias Básicas, Universidad Santiago de Cali, Cali, Colombia; 3 Facultad de Ciencias Exactas Y Aplicadas, Instituto Tecnológico Metropolitano, Medellin, Colombia

**Keywords:** Dental cement, Glycidyl methacrylate, Glass-ionomer, M06, Polyacids

## Abstract

This work is part of a larger study whose main objective was to find a series of promising molecules to be used as glass-ionomer-type materials. The project was divided into 3 successive stages; the results of the first stage have been previously published and were used to continue the study. The molecules evaluated in the second stage were constructed by adding a glycidyl methacrylate molecule to the carboxylic groups of the polyacids selected in the previous stage. The modeling was done using the density functional theory for M06-2X/6-311G(d,p). The results indicate that the addition over the carboxylic groups of the fraction of the molecule, corresponding to itaconic acid, is thermodynamically favored. The final stage was modeled with the M06 functional and consisted of obtaining basic structures of glass-ionomer-type materials, by acid–base reaction between the molecules resulting from the second stage with individual ions of Ca (2 +), Zn (2 +), or Al (+ 3). It was concluded that aluminum atoms generate more compact structures that would correlate with more resistant materials.

**Supplementary Information:**

The online version contains supplementary material available at 10.1007/s00894-022-05211-x.

## Introduction

The invention of dental cements emerged as an aesthetic alternative to the use of materials such as amalgams [1]. However, there is still a need for improved materials that solve problems such as color stability, thermal expansion, low adhesion to the tooth, lack of mechanical resistance, bacterial penetration, material stability, toxicity, setting, and operation times, among others.

After many studies and changes in research, the approach to solve these problems has been focused on materials referred to as glass-ionomers [2–4]. This is a solid matrix obtained from acid–base reaction between polyacid molecule and a metal. In conventional glass ionomer cements (GIC), the metal comes from the degradation of a glass, the polyacid is a polyelectrolyte that includes mono, di, and tricarboxylic acids and water acts as the reaction medium.

Experimentally, polyacids are obtained by free radical polymerization reaction of the monomers of interest. Reaction is carried out in aqueous solution and in the presence of an initiator. Structurally, polyacids are represented by linear chains containing an excess acid groups [5, 6].

It has been suggested to use polymers that are obtained from the combination of different unsaturated carboxylic acids, for example, acrylic acid with itaconic acid and/or maleic acid, with the purpose of increasing the degree of cross-linking within the molecule and therefore culminating in obtaining of materials with greater hardness [7, 8].

During setting, an acid–base reaction occurs between the polyacid and the ionic leachable glass [9, 10]. Polyacids can be modified with compounds that contain the methacrylate group, so that in addition to the acid–base reaction, they undergo a free radical polymerization reaction [5, 11–13]. The setting process occurs in three successive steps [9]. Step 1 is the decomposition of glass powder. Carboxylic acid ionizes and generates H + protons that can subsequently react with the glass surface, releasing Al^3+^, Ca^2+^, Na^+^, F^−^, etc. ions. It is emphasized that not all carboxylic groups are ionized. Then, these ions are precipitated as insoluble polyacrylates. Al^3+^, Ca^2+^, and Zn^2+^ form metal salt bridges with free groups (–COO–) which allows cross-linking of the polycarboxylate chains and also the hardening.

Step 2 describes the precipitation-gelation of cations and anions, and the final step is referred to as the maturation phase [9, 10].

This work presents the continuation of the study published by Gaviria [14] (named as stage 1), where the most stable molecules of some basic structures that make up polyacids were chosen to be evaluated as reagents in obtaining glass-ionomer materials.

The starting polyacid monomers were the following: AACA-1, AADH-1, AADOH-3, ABA-1, AG-1, AGA-2, AH-2, EU-2, MBA-1, MG-1, MGA-1, MH-1, NMP-1, NVC-1, and NVP-1, where AACA is N-acryloyl-6-aminocaproic acid, AADH is molecule derived from phenylalanine, AADOH is molecule derived from tyrosine, ABA is acryloyl β-alanine, AG is acryloyl glycine, AGA is acryloyl glutamic acid, AH is acryloyl histidine, EU is eugenol, MBA is methacryloyl β-alanine, MG is methacryloyl glycine, MGA is methacryloyl glutamic acid, MH is methacryloyl histidine, NMP is N-methacryloyl proline, NVC is N-vinylcaprolactam, and NVP is N-vinylpyrrolidone.

AACA-1 means that the AACA is in the first place of the structure made of AACA, acrylic acid (AA), and itaconic acid (IA). On the other hand, EU-2 means that the Eugenol is found in position 2 of the molecular structure that represents the union between an acrylic acid molecule, a eugenol molecule, and an itaconic acid.

Stages 2 and 3 of the project, reported in this work, consist of the study of the reaction of polyacid monomers with a molecule of glycidyl methacrylate (GM) in some of the carboxyl groups of the substrate, according to the nature of the amino acids.

Finally, the most stable molecules were selected and the way their molecular structure is modified by their union with Ca, Zn, and Al ions, to yield the products of the setting reaction, was analyzed.

## Methods

In this work, the nomenclature, proposed in the article by Gaviria [15], has been followed to identify the different positions for the addition of the GM group to the COO^−^ functional groups. The products of that reaction were modeled at positions 1, 2, 3, and 4.

**Fig. 1:**
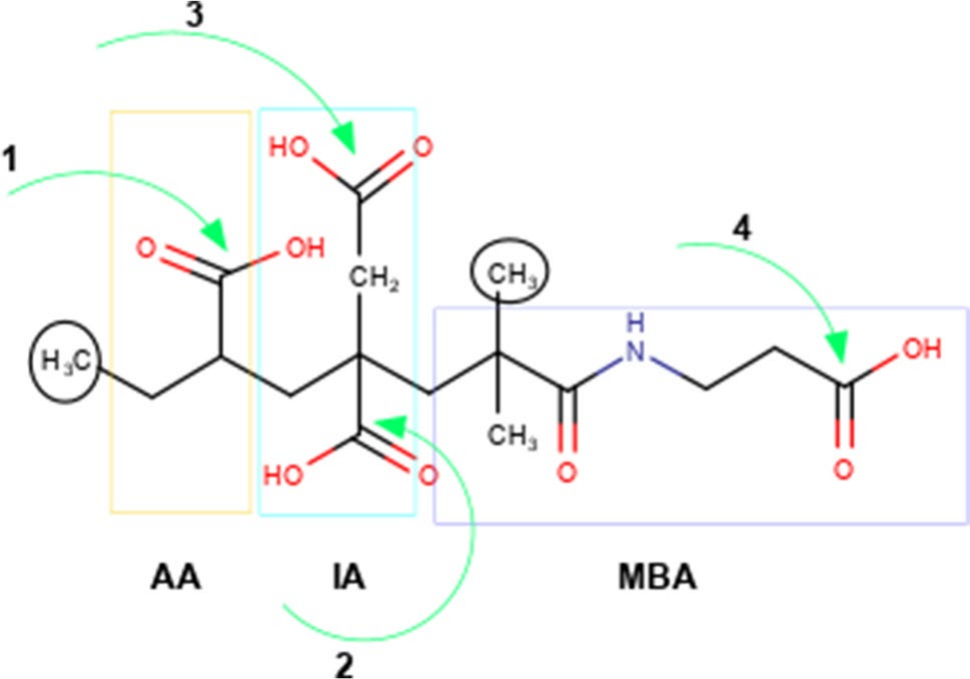
Nomenclature of the acid groups that could react with the GM; the structure of the AA-IA-MBA is shown as an example

Figure 1 lists the COOH groups of the monomer. A base molecule of one of the polyacids is taken as an example. Position 1 corresponds to the COOH group of acrylic acid. Itaconic acid has position 2 that corresponds to the COOH group that is attached to carbon C, and position 3 represents COOH attached to carbon CH_2_. Position 4 refers to a COOH group of the amino acid derivative.

All molecules were optimized in the Gaussian 09 computational package [16], using the density functional theory (DFT). The addition stage of the glycidyl methacrylate to the polyacid (stage 2) was investigated using the global-hybrid meta-GGA M06-2X functional [17] with the 6-311G(d,p) basis set [18].

In the optimization of the glass ionomer molecules (stage 3), the M06 functional [17] and the 6-311G(d,p) basis set were used. That functional has been parametrized including both transition metals and nonmetals.

To build the molecular structures of conventional glass ionomers (GICs), the local indices of the condensed Fukui functions (${f}^{-}$) [19–21] were obtained using the finite difference method aiming to find the COOH groups with the highest electrophilic character that preferentially interact with the Zn, Ca, or Al cations.

The Hirshfeld charges [19] for the atoms in the most stable molecules obtained from the reaction of the polyacid with the GM group were calculated using the Multiwfn program [22]. Hirshfeld charges are recommended for the calculation of condensed Fukui functions [22, 23].

The condensed Fukui function for an electrophilic attack on atom A (${f}_{A}^{-}$) can be defined as follows:

1 $${f}_{A}^{-}={\rho }_{N}^{A}-{\rho }_{N-1}^{A}$$

where *ρ*^*A*^ is the electronic population of atom A. Taking into account that the atomic charge of A is defined as follows:

2 $${q}^{A}={Z}^{A}-{\rho }^{A}$$

(*Z* is the charge of the atomic nucleus), Eq. 1 can be rewritten as follows:

3 $${f}_{A}^{-}={q}_{N}^{A}-{q}_{N-1}^{A}$$

Geometry optimizations were followed by analytical calculations of frequencies to determine the nature of the stationary point and obtain the thermodynamic parameters of the molecules. To calculate enthalpy an entropy at a temperature *T*, the difference between the values at that temperature and 0 K has been evaluated according to standard thermodynamics equations, they assume non-interacting particles. A harmonic oscillator model has also been used [24].

Thermal corrections to enthalpy and entropy values have been evaluated at the experimental temperatures (313.15 K and 298 K) to stage 2 and stage 3, respectively, and 1 atm pressure.

Enthalpy, entropy, and free energy are calculated from the partition function with contributions from translation, electronic, rotational, and vibrational motion.

Vibrational frequency calculations were performed at M06/6-311G(d,p) for modified polyacids with glycidyl methacrylate (stage 2). Optimizations of Ca, Zn, and Al atoms were performed under the same conditions.

The effect of the aqueous solution on all the molecules studied was carried out using the polarizable continuous model, specifically the integral equation formalism (IEFPCM) [25].

## Results

### Addition of glycidyl methacrylate to the polyacid (stage 2)

**Fig. 2:**
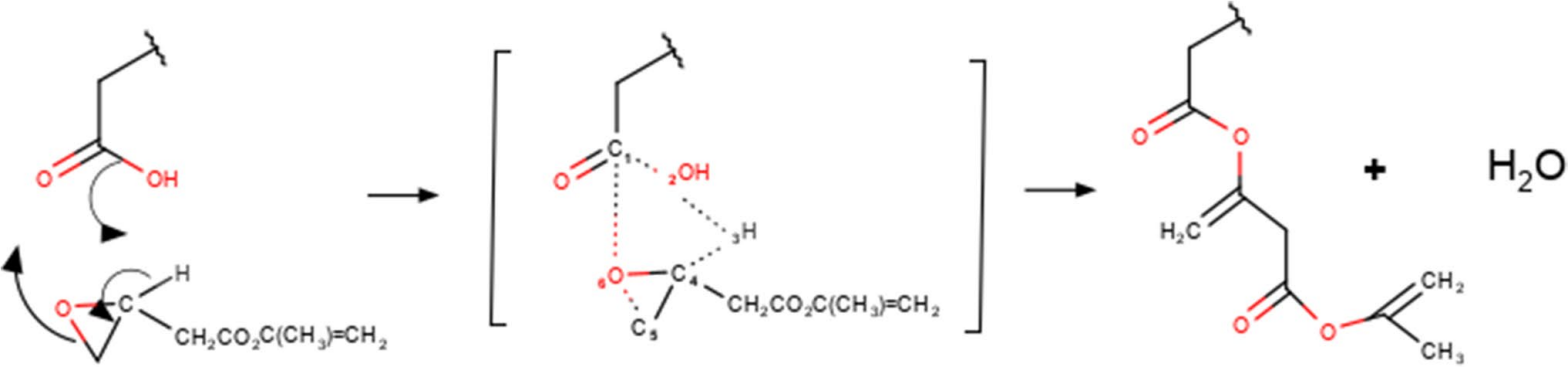
Reaction mechanism between the polyacid and GM

The GM addition reaction to the substrate is thought to occur according to the process depicted in Fig. 2 [15]. The mechanism involves a transition state of six members: The hydroxyl group of the carboxylic acid (OH) binds H-3 to form a water molecule.

The results obtained from the calculations in stage 2 are reported in Table 1. Table S1 in the electronic supplementary material shows the energy data of the studied molecules and the optimized molecular structures can be seen in Figure S1.

**Table 1:** Relative energy (Δ*G*) of the glycidyl methacrylate addition reaction products

Molecule	Δ*G* (kJ mol^−1^)	Molecule	Δ*G* (kJ mol^−1^)	Molecule	Δ*G* (kJ mol^−1^)	Molecule	Δ*G* (kJ mol^−1^)
AACA-1-GM1	92.8	ABA-1-GM4	47.5	EU-2-GM3	0.0	MH-1-GM2	11.4
AACA-1-GM2	20.2	AG-1-GM1	0.0	EU-2-GM4**	–	MH-1-GM3	0.0
AACA-1-GM3	0.0	AG-1-GM2	1.6	MBA-1-GM1	85.1	MH-1-GM4	88.6
AACA-1-GM4	89.1	AG-1-GM3	5.8	MBA-1-GM2	0.0	NMP-1-GM1	56.1
AADH-1-GM1	37.6	AG-1-GM4	21.7	MBA-1-GM3	22.1	NMP-1-GM2	0.0
AADH-1-GM2	81.3	AGA-2-GM1	60.2	MBA-1-GM4	62.01	NMP-1-GM3	12.5
AADH-1-GM3	0.0	AGA-2-GM2	0.0	MG-1-GM1	0.0	NMP-1-GM4	82.5
AADH-1-GM4	13.3	AGA-2-GM3	6.4	MG-1-GM2	28.0	NVC-1-GM1	107.44
AADOH-3-GM1	59.7	AGA-2-GM4*	75.9 (63.5)	MG-1-GM3	56.2	NVC-1-GM2	0.0
AADOH-3-GM2	7.3	AH-21-GM1	4.7	MG-1-GM4	57.9	NVC-1-GM3	75.6
AADOH-3-GM3	0.0	AH-2-GM2	0.0	MGA-1-GM1	16.6	NVC-1-GM4**	–
AADOH-3-GM4	150.4	AH-2-GM3	5.5	MGA-1-GM2	0.0	NVP-1-GM1	6.2
ABA-1-GM1	39.2	AH-2-GM4	142.8	MGA-1-GM3	29.8	NVP-1-GM2	0.0
ABA-1-GM2	0.0	EU-2-GM1	25.8	MGA-1-GM4^*^	145.2 (31.9)	NVP-1-GM3	12.9
ABA-1-GM3	8.4	EU-2-GM2	28.1	MH-1-GM1	19.7	NVP-1-GM4**	–

*Two COOH groups in the molecule of the amino acid derivative that forms the polyacid.

**There are no COOH groups in the structure of the amino acid derivative.

For example, AACA-1-GM2 refers to the polyacid AACA-AA-IA for which the glycidyl methacrylate group has been attached to the carbon at position 2 and AACA-1-GM1 when the addition of that group occurs on the carbon in position 1 (see Fig. 1).

The relative free energy values (ΔG) indicate that the most stable molecules in the GM group addition reaction are as follows: AACA-1-GM3, AADH-1-GM3, AADOH-3-GM3, ABA-1-GM2, AG-1-GM1, AGA-2-GM2, AH-2-GM2, EU-2-GM3, MBA-1-GM2, MG-1-GM1, MGA-1-GM2, MH-1-GM3, NMP -1-GM2, NVC-1-GM2, and NVP-1-GM2.

The results express that approximately 53% of the molecules studied are more stable when the addition of the GM group occurs on the carboxyl group attached to carbon “C” of itaconic acid (position 2), and ≈ 33% of the molecules are more stable when the addition of GM occurs on the carboxyl bonded to the “CH_2_” carbon also of itaconic acid (position 3). The remaining percentage (≈13%) of the molecules studied have less Gibbs energy, if the addition occurs in the carboxyl group of acrylic acid (position 1).

**Fig. 3:**
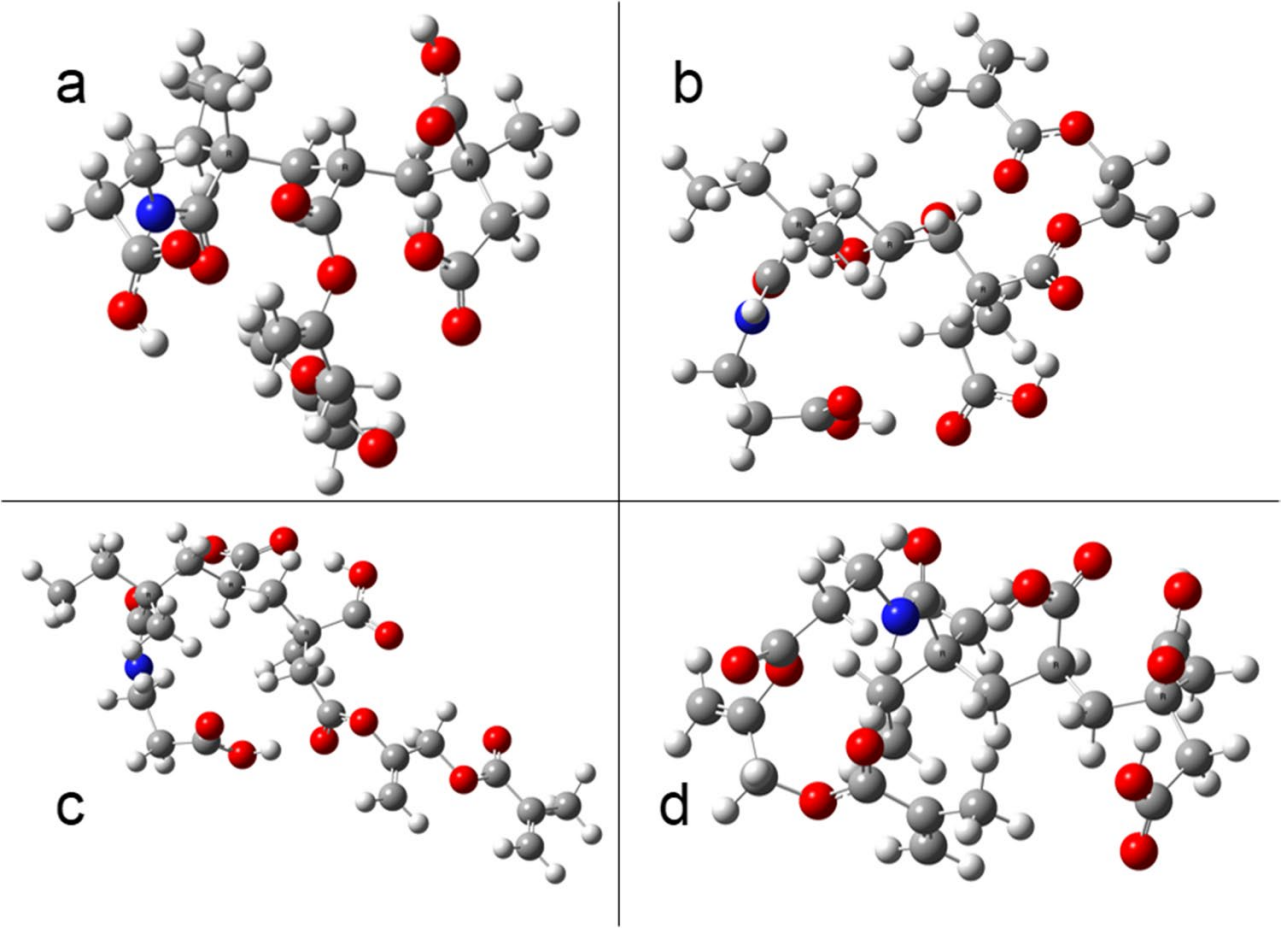
Optimized molecular structure of the reaction product of MBA-1 with GM in the different carboxyl groups. **a** Position 1, **b** position 2, **c** position 3, **d** position 4

For example, the AADOH-3-GM4 and AH-2-GM4 molecules present higher energy values than AADOH-3-GM3 and AH-2-GM2, up to 150 and 143 kJ mol^**−1**^, respectively. Figure 3 shows the optimized structures of the molecules resulting from the addition of GM to the polyacid MBA-1.

Thermodynamically, the addition of the GM group has a preference over the carboxyl groups of itaconic acid.

The data show that most of the molecules present the lowest values of electronic energy (the data are collected in the electronic supplementary material) when the GM group is added in positions 2 or 3; 60% of the molecules analyzed have lower free energy because they also have lower enthalpy. It is concluded that positions 2 and 3 allow favorable atomic interactions and reduce unfavorable interactions.

When the addition of GM occurs in position 1, a greater number of molecules have higher entropy values, meaning that the entropic component is favored but at the cost of losing favorable atomic interactions.

### Setting reaction for the formation of the glass-ionomer (stage 3)

**Fig. 4:**
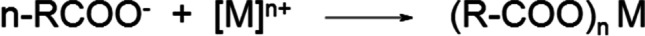
General representation of the acid–base reaction for the formation of the basic structure of the glass ionomer; M: Ca, Zn, Al; *n* = 2 or 3

Based on the results obtained from stage 2, we proceeded to model the structures resulting from the reaction of the selected molecules with the cations of Zn, Ca and Al (glass components), by an acid–base reaction, which is represented in Fig. 4.

Hirshfeld atomic charges and Fukui indices calculated for each atom are given in Table S2 in the electronic supplementary material.

Table 2 shows the average bond distances of the metal with the atoms to which it is attached and the entropies of each optimized molecule.

**Table 2:** Bond distances around the metal (Å) and entropy values for the molecule (cal.mol^-1^K^-1^) obtained from the molecular modeling of M06/6-311G(d,p)

Molecule		Average distance (Å)/entropy (cal.mol^-1^K^-1^)	Molecule		Average distance (Å)/entropy (cal.mol^-1^K^-1^)	Molecule		Average distance (Å)/entropy (cal.mol^-1^K^-1^)
AACA-1-GM3	Ca	2.46/240	AGA-2-GM2	Ca	2.43/235	MGA-1-GM2	Ca	2.26/244
	Zn	1.92/233		Zn	2.02/233		Zn	1.89/238
	Al	1.82/220		Al	1.82/230		Al	1.77/230
AADH-1-GM3	Ca	2.32/256	AH-2-GM2	Ca	2.41/247	MH-1-GM3	Ca	2.48/247
	Zn	1.91/255		Zn	1.97/237		Zn	1.95/241
	Al	1.84/247		Al	2.06/229		Al	1.76/240
AADOH-3-GM3	Ca	2.38/256	EU-2-GM3	Ca	2.33/229	NMP-1-GM2	Ca	2.35/222
	Zn	2.07/254		Zn	1.83/231		Zn	1.90/230
	Al	1.73/256		Al	1.74/230		Al	1.86/243
ABA-1-GM2	Ca	2.39/220	MBA-1-GM2	Ca	2.44/233	NVC-1-GM2	Ca	2.16/229
	Zn	1.96/224		Zn	1.92/226		Zn	1.86/222
	Al	1.80/217		Al	1.80/221		Al	1.69/217
AG-1-GM1	Ca	2.22/224	MG-1-GM1	Ca	2.29/224	NVP-1-GM2	Ca	2.39/203
	Zn	1.89/221		Zn	1.89/224		Zn	1.93/202
	Al	1.76/207		Al	1.72/208		Al	1.76/198

From the average bond distances, it can be seen that when the atom involved is calcium, the values obtained are between 2.19 and 2.56 Å; when the metal is replaced by Zn or Al, the average values are in the ranges of 1.83–2.07 and 1.69–2.06 Å, respectively. In addition, molecular geometry shows that calcium and aluminum atoms make 4 bonds with neighboring atoms in almost all of the optimized molecules, while in those molecules that involve the zinc atom, 2 bonds with neighboring atoms are observed.

The entropy values for each molecule show that in most of them (approximately 87%), the lowest value is for the molecules that have the Al atom, and in 80% of the molecules, the highest molecular entropy value occurs when they contain the Ca atom.

The results of the molecular entropy are in agreement with the data obtained from the bond distances. The data indicate that the aluminum atom generates a molecular contraction around it, that is, it promotes greater and stronger interactions, evidenced by lower bond distances around it, a greater number of interactions and a lower value of molecular entropy, when the same molecules with a different metal atom are compared.

Materials with the Al atom would be expected to be more compact and therefore would correlate with materials of higher density, higher hardness, and higher strength.

**Fig. 5:**
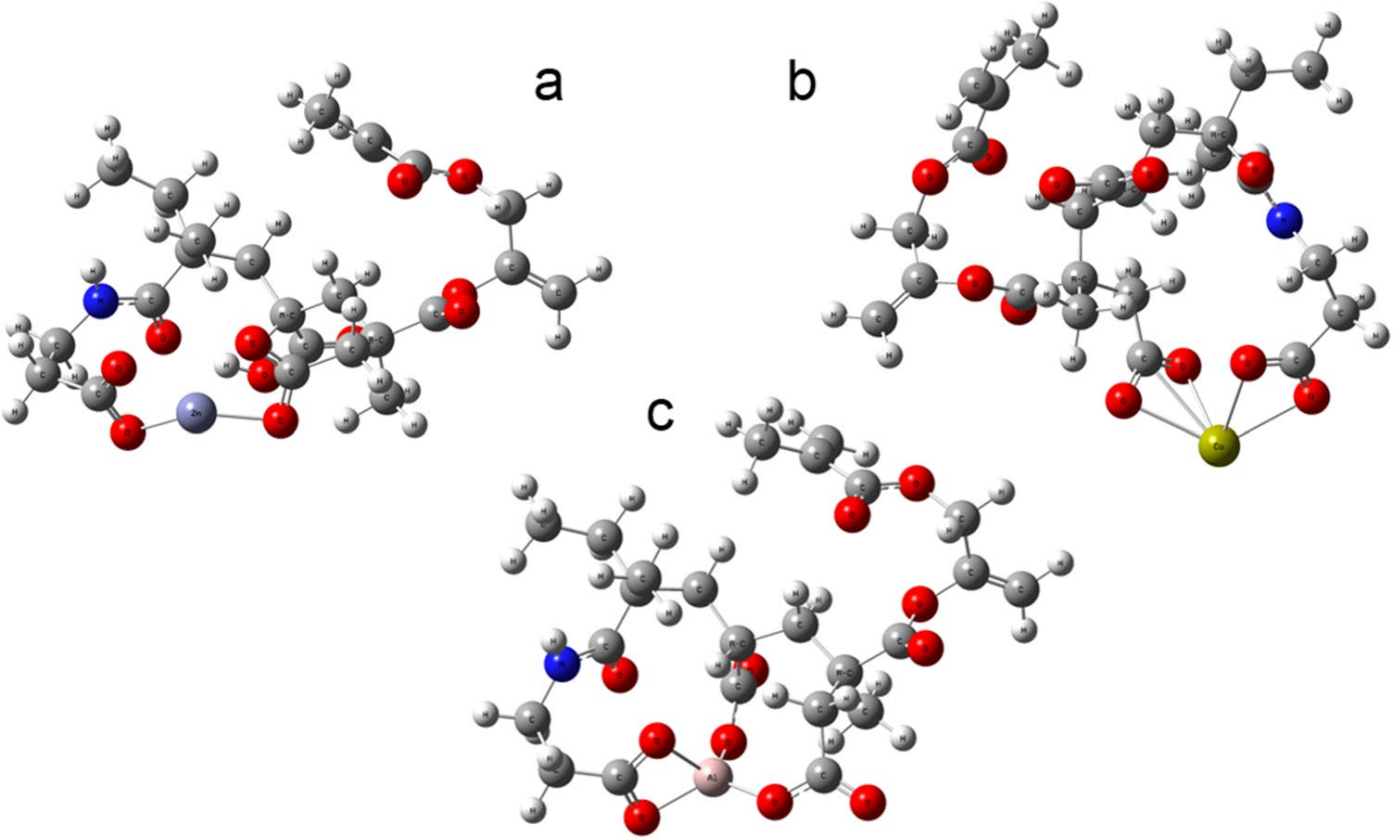
Molecules optimized to M06/6-311G(d,p) of MBA-1-GM1 with cations. **a** Zn, **b** Ca, **c** Al

Figure 5 provides a representation of the interactions that occur with the different metals and the branched polyacid.

We have also determined the interaction energy of the metal with the polymer, in order to quantify the possible stabilization or not that occurs in the molecule.

It was calculated as Gibb energy difference of the GIC molecules and the modified polymers and respective metals, that is:

4 $${E}_{i}={E}_{GIC}-{E}_{P}-{E}_{M}$$

where ${E}_{i}$, interaction energy; ${E}_{GIC}$, glass ionomer conventional energy; ${E}_{P}$, modified polymer energy; and ${E}_{M}$, metal energy (Ca, Zn, or Al).

The results are compiled in Table 3. Each energy component used in Eq. (4) can be found in Table S3.

**Table 3:** ${E}_{i}$**:** Interaction energy (Hartree) between modified polymer and metal atom. Calculated to M06/6-311G(d,p)

Molecule	Metal	${E}_{i}$	Molecule	Metal	${E}_{i}$	Molecule	Metal	${E}_{i}$
AACA-1-GM3	Ca	0.965729	AGA-2-GM2	Ca	0.944139	MGA-1-GM2	Ca	0.987918
	Zn	1.125804		Zn	1.120595		Zn	1.140861
	Al	1.558237		Al	1.550672		Al	1.566133
AADH-1-GM3	Ca	0.964736	AH-2-GM2	Ca	0.95241	MH-1-GM3	Ca	0.963901
	Zn	1.161891		Zn	1.139624		Zn	1.140749
	Al	1.613027		Al	1.588899		Al	1.539402
AADOH-3-GM3	Ca	0.966217	EU-2-GM3	Ca	0.944463	NMP-1-GM2	Ca	0.979992
	Zn	1.168629		Zn	1.139819		Zn	1.146478
	Al	1.721253		Al	1.220413		Al	1.696044
ABA-1-GM2	Ca	0.962179	MBA-1-GM2	Ca	0.963159	NVC-1-GM2	Ca	1.001583
	Zn	1.134088		Zn	1.131577		Zn	1.146076
	Al	1.576354		Al	1.567417		Al	1.291851
AG-1-GM1	Ca	0.972577	MG-1-GM1	Ca	0.969037	NVP-1-GM2	Ca	0.967644
	Zn	1.147477		Zn	1.149828		Zn	1.136887
	Al	1.597945		Al	1.654396		Al	1.145604

From Table 3, in principle, it can be seen that the interaction energy is positive for all the polymers modified with GM and with each of the Ca, Zn, and Al metals.

That is, GICs are less stable than the respective polymers without undergoing acid–base reaction. It was also found that the greatest molecular destabilization occurs in the interaction of each of the polymers with aluminum and that the Ca atom generates the most stable molecules.

For example, average interaction energy values of 1.5, 1.1, and 0.97 Hartree are observed when the metal is Al, Zn, and Ca, respectively.

The energetic results obtained are in agreement with the compactness of the structures, previously analyzed with the bond distances and the entropic component.

## Conclusions

A series of promising molecules were computationally evaluated to be used as glass-ionomer-type materials, built in 3 successive stages. This article presented the results obtained from the last 2 stages of the study. The results of the addition of glycidyl methacrylate to the polyacid indicate that the most stable molecules are those generated by the addition to the carboxylic groups of the fraction of the molecule corresponding to itaconic acid; there, the enthalpy component prevails over the entropic unfavorability.

The basic structures of the glass-ionomers, composed of the most stable molecules obtained from the addition of GM to the studied polyacids and individual Ca (2^+^), Zn (2^+^), and Al (3^+^) ions, were optimized.

The results from the molecular optimization allow us to conclude that the aluminum atom allows a greater compaction of the structure, evidenced in the number of atomic bonds it forms, the distances of the bonds, and the total entropy of the molecule. Although, they are also structured with less relative stability.

## Supplementary Information

Below is the link to the electronic supplementary material.

Supplementary file1 (PDF 703 KB)

## Data Availability

All data generated or analyzed during this study are included in this published article (and its supplementary information file).
