# Supplementary material for: Computational study of the relative stability of some glass-ionomer cement-forming molecules
Source: J Mol Model. 2022 Sep 28;28(10):333. doi: 10.1007/s00894-022-05211-x (PMC9519700; doi:10.1007/s00894-022-05211-x)
Supplement: Supplementary file 1 — Supplementary file1 (PDF 703 KB) [file 894_2022_5211_MOESM1_ESM.pdf]

Journal of Molecular Modeling

Supplementary Information

Computational study of the relative stability of some glass-ionomer cement-forming molecules.

Jair Gaviria[1*], Silvia Quijano[2], Jairo Quijano[1], Pablo Ruiz[1,3]

[1] Laboratorio de Fisicoquímica Orgánica, Facultad de Ciencias, Universidad Nacional de Colombia, Medellín, Colombia, 050034

[2] Programa de Microbiología, Facultad de Ciencias Básicas, Universidad Santiago de Cali, Cali, Colombia

[3] Facultad de Ciencias Exactas y Aplicadas, Instituto Tecnológico Metropolitano, Medellín, Colombia

*jgarango@unal.edu.co

https://orcid.org/0000-0003-0293-7078

Table S1. Electronic Energies (Eel), zero-point vibrational energies (ZPE), thermal correction to enthalpies (TCH), enthalpies (H) and Gibbs energies (G) in Hartrees; entropies (S) in cal·mol^{-1}·K^{-1}, for all the molecules as products of the addition reaction of glycidyl methacrylate (GM) to the polyacids studied. Calculated at M06-2X/6-311G(d,p) at 313.15 K.

Molecule	GM Position	Eel	ZPE	TCH	S	H	G
**AACA-1**	1	-1896.34044	0.650811	0.694893	244.981	-1895.65	-1895.7678
	2	-1896.368707	0.650118	0.694441	242.862	-1895.67	-1895.7955
	3	-1896.376103	0.649998	0.694478	243.529	-1895.68	-1895.8032
	4	-1896.339988	0.650404	0.695238	249.44	-1895.64	-1895.7692
**AH-2**	1	-2003.438934	0.614876	0.660193	253.969	-2002.78	-2002.9055
	2	-2003.44223	0.615273	0.660341	251.216	-2002.78	-2002.9073
	3	-2003.444175	0.615524	0.659873	242.207	-2002.78	-2002.9052
	4	-2003.387192	0.614124	0.659035	249.931	-2002.73	-2002.8529
**AADH-1**	1	-2048.739667	0.673556	0.720579	254.529	-2048.02	-2048.1461
	2	-2048.716184	0.672517	0.71975	266.541	-2048	-2048.1294
	3	-2048.744561	0.67241	0.720096	272.418	-2048.02	-2048.1604
	4	-2048.746397	0.672633	0.719175	256.74	-2048.03	-2048.1553
**MH-1**	1	-2042.7399	0.643247	0.689691	258.109	-2042.05	-2042.179
	2	-2042.749009	0.643463	0.689015	244.839	-2042.06	-2042.1822
	3	-2042.749674	0.644272	0.690349	254.866	-2042.06	-2042.1865
	4	-2042.713358	0.643413	0.689758	258.796	-2042.02	-2042.1527
**NVP-1**	1	-1627.955671	0.554806	0.592718	216.874	-1627.36	-1627.4712
	2	-1627.958087	0.554438	0.592705	216.701	-1627.37	-1627.4735
	3	-1627.951336	0.554311	0.592665	220.313	-1627.36	-1627.4686
		There are no COOH groups at the amino acid position					
**NMP-1**	1	-1895.124967	0.627608	0.670979	241.863	-1894.45	-1894.5747
	2	-1895.145252	0.627241	0.670713	243.486	-1894.47	-1894.596
	3	-1895.141165	0.627642	0.670875	242.423	-1894.47	-1894.5913
	4	-1895.117622	0.628543	0.671691	237.853	-1894.45	-1894.5646
**NVC-1**	1	-1706.534095	0.612147	0.652587	228.443	-1705.88	-1705.9955
	2	-1706.574889	0.612946	0.653339	230.147	-1705.92	-1706.0364
	3	-1706.545445	0.614081	0.654632	234.036	-1705.89	-1706.0076
		There are no COOH groups at the amino acid position					
**EU-2**	1	-1802.58143	0.609124	0.652276	248.555	-1801.93	-1802.0532
	2	-1802.589327	0.610579	0.652703	231.866	-1801.94	-1802.0523
	3	-1802.596847	0.608914	0.651743	236.29	-1801.95	-1802.063
		There are no COOH groups at the amino acid position					
**ABA-1**	1	-1778.422643	0.562605	0.604247	237.814	-1777.82	-1777.9371
	2	-1778.441805	0.563353	0.604274	229.37	-1777.84	-1777.952
	3	-1778.436145	0.563322	0.604518	234.772	-1777.83	-1777.9488
	4	-1778.423053	0.563731	0.604289	230.731	-1777.82	-1777.9339
**MBA-1**	1	-1817.719451	0.591469	0.634093	239.29	-1817.09	-1817.2048

	2	-1817.75288	0.592265	0.634228	237.489	-1817.12	-1817.2372
	3	-1817.743332	0.591071	0.632494	236.262	-1817.11	-1817.2287
	4	-1817.730334	0.59158	0.633639	234.158	-1817.1	-1817.2135
**AADOH-3**	1	-2123.952656	0.677866	0.725783	254.479	-2123.23	-2123.3539
	2	-2123.97071	0.677982	0.725802	258.29	-2123.24	-2123.3738
	3	-2123.969976	0.677108	0.725805	265.347	-2123.24	-2123.3766
	4	-2123.913945	0.677835	0.726375	263.947	-2123.19	-2123.3193
**AG-1**	1	-1739.118093	0.53415	0.574118	229.631	-1738.54	-1738.6586
	2	-1739.119103	0.533761	0.573435	225.036	-1738.55	-1738.658
	3	-1739.118017	0.53383	0.57335	223.796	-1738.54	-1738.6563
	4	-1739.109131	0.53373	0.573485	229.747	-1738.54	-1738.6503
**AGA-2**	1	-2006.311762	0.609282	0.653701	244.276	-2005.66	-2005.78
	2	-2006.327976	0.607101	0.652571	255.488	-2005.68	-2005.8029
	3	-2006.331025	0.607935	0.652377	244.122	-2005.68	-2005.8005
	4 (CH)	-2006.299016	0.606954	0.652452	255.388	-2005.65	-2005.774
	4(CH2)	-2006.308899	0.608522	0.65307	246.263	-2005.66	-2005.7787
**MGA-1**	1	-2045.605403	0.635343	0.681602	258.832	-2044.92	-2045.053
	2	-2045.616947	0.636361	0.68269	250.524	-2044.93	-2045.0593
	3	-2045.605807	0.635699	0.681514	247.754	-2044.92	-2045.0479
	4 CH	-2045.562104	0.634798	0.680198	244.572	-2044.88	-2045.004
	4 CH2	-2045.604195	0.636062	0.682095	250.51	-2044.92	-2045.0471
**MG-1**	1	-1778.425774	0.561651	0.603336	240.338	-1777.82	-1777.9424
	2	-1778.42237	0.563111	0.603922	226.98	-1777.82	-1777.9317
	3	-1778.410302	0.561952	0.602779	227.356	-1777.81	-1777.921
	4	-1778.412814	0.562927	0.603219	221.892	-1777.81	-1777.9203

Table S2. Hirshfeld atomic charges, cation (qN-1), neutral (qN) and condensed Fukui indices (f^-). See figures S1 for numbering.

AACA-1-GM3					AADH-1-GM3				
#	atom	qN-1	qN	(f^-)	#	atom	qN-1	qN	(f^-)
1	C	0,012954	0,012616	-0,000338	1	C	-0.015356	-0.017181	-0.001825
2	C	-0,046663	-0,048989	-0,002326	2	C	-0.008526	-0.009056	-0.00053
3	H	0,030253	0,028338	-0,001915	3	H	0.023513	0.024129	0.000616
4	H	0,043386	0,032891	-0,010495	4	C	-0.077643	-0.08249	-0.004847
5	C	-0,034618	-0,033775	0,000843	5	H	0.041576	0.034792	-0.006784
6	H	0,00706	0,018773	0,011713	6	H	0.040634	0.035021	-0.005613
7	C	0,208372	0,205249	-0,003123	7	H	0.037412	0.031748	-0.005664
8	O	-0,253317	-0,280517	-0,0272	8	C	0.197982	0.189744	-0.008238
9	O	-0,171684	-0,183931	-0,012247	9	O	-0.214154	-0.211343	0.002811
10	H	0,138239	0,140189	0,00195	10	N	-0.060446	-0.067122	-0.006676
11	C	-0,04153	-0,046811	-0,005281	11	H	0.150264	0.13655	-0.013714
12	H	0,052277	0,033836	-0,018441	12	C	0.046573	0.03256	-0.014013
13	H	0,038364	0,031626	-0,006738	13	C	-0.028765	-0.045692	-0.016927
14	C	-0,018692	-0,022351	-0,003659	14	H	0.053952	0.031537	-0.022415
15	H	0,04092	0,030403	-0,010517	15	H	0.071926	0.043217	-0.028709
16	C	0,215308	0,174241	-0,041067	16	C	0.114156	-0.015355	-0.129511
17	O	-0,123327	-0,287524	-0,164197	17	C	0.115027	-0.038942	-0.153969
18	N	0,114825	-0,073842	-0,188667	18	C	0.015659	-0.047961	-0.06362
19	H	0,176532	0,121756	-0,054776	19	C	0.021546	-0.044169	-0.065715
20	C	0,031647	-0,001636	-0,033283	20	C	0.034723	-0.030231	-0.064954
21	H	0,071741	0,028348	-0,043393	21	C	0.026952	-0.039939	-0.066891
22	H	0,085883	0,033995	-0,051888	22	H	0.075923	0.038751	-0.037172
23	C	-0,045192	-0,051098	-0,005906	23	H	0.053869	0.021833	-0.032036
24	H	0,034273	0,023815	-0,010458	24	H	0.089971	0.05088	-0.039091
25	H	0,044474	0,030896	-0,013578	25	H	0.085906	0.044323	-0.041583
26	C	-0,038035	-0,045168	-0,007133	26	H	0.099421	0.044681	-0.05474
27	H	0,040588	0,031737	-0,008851	27	C	-0.045084	-0.046223	-0.001139
28	H	0,047815	0,030279	-0,017536	28	H	0.042203	0.034107	-0.008096
29	C	-0,041234	-0,044517	-0,003283	29	H	0.03295	0.032074	-0.000876
30	H	0,050626	0,035693	-0,014933	30	C	-0.028977	-0.027291	0.001686
31	H	0,031661	0,033162	0,001501	31	H	0.013964	0.023624	0.00966
32	C	-0,04608	-0,049445	-0,003365	32	C	-0.050435	-0.052336	-0.001901
33	H	0,061483	0,05154	-0,009943	33	H	0.032994	0.02711	-0.005884
34	H	0,05011	0,04208	-0,00803	34	H	0.031882	0.026671	-0.005211
35	C	0,2289	0,231012	0,002112	35	C	0.202794	0.204837	0.002043
36	O	-0,222791	-0,233223	-0,010432	36	O	-0.254442	-0.271135	-0.016693
37	O	-0,149907	-0,143239	0,006668	37	O	-0.187687	-0.179555	0.008132
38	H	0,133817	0,136815	0,002998	38	H	0.120239	0.125882	0.005643
39	C	-0,049936	-0,052413	-0,002477	39	C	0.014964	0.015127	0.000163
40	H	0,053112	0,04628	-0,006832	40	C	0.212313	0.207364	-0.004949
41	H	0,058656	0,052227	-0,006429	41	O	-0.27797	-0.252972	0.024998
42	C	0,235249	0,234938	-0,000311	42	O	-0.171715	-0.182036	-0.010321
43	O	-0,226063	-0,213305	0,012758	43	H	0.128928	0.123846	-0.005082
44	O	-0,086756	-0,094037	-0,007281	44	C	-0.048207	-0.050905	-0.002698
45	C	-0,036717	-0,04407	-0,007353	45	H	0.057688	0.054151	-0.003537
46	H	0,052219	0,031177	-0,021042	46	H	0.048978	0.04472	-0.004258
47	H	0,042524	0,033534	-0,00899	47	C	-0.085647	-0.084989	0.000658
48	C	-0,082681	-0,089008	-0,006327	48	H	0.042306	0.038594	-0.003712
49	H	0,055541	0,033564	-0,021977	49	H	0.017983	0.031143	0.01316
50	H	0,022245	0,026498	0,004253	50	H	0.032196	0.027315	-0.004881
51	H	0,034286	0,026405	-0,007881	51	C	0.233625	0.232782	-0.000843
52	C	-0,073402	-0,078922	-0,00552	52	O	-0.210406	-0.214009	-0.003603
53	H	0,042127	0,035067	-0,00706	53	O	-0.10124	-0.103529	-0.002289
54	H	0,046968	0,038064	-0,008904	54	C	-0.074923	-0.079298	-0.004375
55	H	0,040096	0,036304	-0,003792	55	H	0.042666	0.040779	-0.001887
56	C	0,218522	0,211093	-0,007429	56	H	0.041922	0.03932	-0.002602
57	O	-0,288196	-0,271524	0,016672	57	H	0.049405	0.038953	-0.010452
58	O	-0,166943	-0,181308	-0,014365	58	H	0.037512	0.031505	-0.006007

59	H	0,128608	0,125889	-0,002719
60	C	0,072688	0,073855	0,001167
61	C	-0,068712	-0,083687	-0,014975
62	H	0,060721	0,052255	-0,008466
63	H	0,065055	0,058685	-0,00637
64	C	0,029631	0,02853	-0,001101
65	H	0,032936	0,036992	0,004056
66	H	0,056497	0,04978	-0,006717
67	O	-0,116264	-0,11742	-0,001156
68	C	0,21178	0,210589	-0,001191
69	C	0,006085	-0,008101	-0,014186
70	C	-0,032698	-0,056427	-0,023729
71	H	0,059087	0,048007	-0,01108
72	H	0,046428	0,040557	-0,005871
73	C	-0,081055	-0,086861	-0,005806
74	H	0,045505	0,03758	-0,007925
75	H	0,047158	0,037226	-0,009932
76	H	0,035157	0,032807	-0,00235
77	O	-0,247894	-0,254026	-0,006132

59	C	0.199794	0.200001	0.000207
60	O	-0.249732	-0.279146	-0.029414
61	O	-0.177266	-0.1871	-0.009834
62	H	0.120214	0.121284	0.00107
63	H	0.069602	0.050894	-0.018708
64	C	0.068624	0.068608	-1.6E-05
65	C	-0.066964	-0.068783	-0.001819
66	H	0.066069	0.061442	-0.004627
67	H	0.050044	0.052577	0.002533
68	C	0.033547	0.032943	-0.000604
69	H	0.05006	0.050026	-3.4E-05
70	H	0.055349	0.052154	-0.003195
71	O	-0.124759	-0.123815	0.000944
72	C	0.208381	0.208502	0.000121
73	C	-0.011588	-0.010086	0.001502
74	C	-0.049825	-0.054895	-0.00507
75	H	0.05168	0.049476	-0.002204
76	H	0.05135	0.048283	-0.003067
77	C	-0.08601	-0.085811	0.000199
78	H	0.04119	0.039132	-0.002058
79	H	0.039681	0.039201	-0.00048
80	H	0.033009	0.036462	0.003453
81	O	-0.265273	-0.267192	-0.001919

		**AADOH-3-GM3**		
#	atom	qN-1	qN	(f^-)
1	H	0.041313	0.032017	-0.009296
2	C	-0.079802	-0.084084	-0.004282
3	H	0.031883	0.029253	-0.00263
4	H	0.033106	0.030613	-0.002493
5	C	-0.037319	-0.040014	-0.002695
6	H	0.03571	0.031802	-0.003908
7	H	0.033574	0.029694	-0.00388
8	C	-0.025021	-0.023884	0.001137
9	H	0.025828	0.025578	-0.00025
10	C	0.217627	0.219182	0.001555
11	O	-0.261833	-0.257126	0.004707
12	O	-0.152802	-0.155743	-0.002941
13	H	0.174953	0.17529	0.000337
14	C	-0.060964	-0.061283	-0.000319
15	H	0.01633	0.017499	0.001169
16	H	0.037866	0.030813	-0.007053
17	C	0.01713	0.016742	-0.000388
18	C	0.213806	0.214156	0.00035
19	O	-0.235378	-0.24824	-0.012862
20	O	-0.156504	-0.157044	-0.00054
21	C	-0.065168	-0.061387	0.003781
22	H	0.019266	0.02993	0.010664
23	H	0.028334	0.030149	0.001815
24	C	0.206439	0.213613	0.007174
25	O	-0.233696	-0.232035	0.001661
26	O	-0.093299	-0.09434	-0.001041
27	C	-0.003945	-0.004401	-0.000456
28	C	-0.012967	-0.013588	-0.000621
29	C	0.187843	0.183341	-0.004502
30	O	-0.229252	-0.246148	-0.016896
31	N	-0.077993	-0.070918	0.007075
32	H	0.113179	0.115771	0.002592
33	C	0.03274	0.024844	-0.007896
34	C	-0.035532	-0.049712	-0.01418
35	H	0.057033	0.038368	-0.018665
36	H	0.067639	0.045237	-0.022402

		**AGA-2-GM2**		
#	atom	qN-1	qN	(f^-)
1	H	0,03404	0,03045	-0,00359
2	C	-0,081084	-0,086092	-0,005008
3	H	0,032288	0,029823	-0,002465
4	H	0,043685	0,032676	-0,011009
5	C	-0,042337	-0,045809	-0,003472
6	H	0,037402	0,030618	-0,006784
7	H	0,034753	0,030706	-0,004047
8	C	-0,019742	-0,021564	-0,001822
9	H	0,037229	0,034598	-0,002631
10	C	0,220581	0,220037	-0,000544
11	O	-0,267979	-0,273514	-0,005535
12	O	-0,158475	-0,160641	-0,002166
13	H	0,150147	0,14852	-0,001627
14	C	-0,039787	-0,042497	-0,00271
15	H	0,046919	0,03438	-0,012539
16	H	0,029405	0,027559	-0,001846
17	C	-0,027127	-0,031745	-0,004618
18	C	0,194212	0,166806	-0,027406
19	O	-0,185611	-0,295066	-0,109455
20	N	0,035889	-0,092143	-0,128032
21	H	0,122754	0,093705	-0,029049
22	C	0,045001	0,029123	-0,015878
23	C	-0,029405	-0,03629	-0,006885
24	H	0,060304	0,042243	-0,018061
25	H	0,05479	0,045848	-0,008942
26	C	-0,048938	-0,054042	-0,005104
27	H	0,042314	0,034434	-0,00788
28	H	0,066993	0,050754	-0,016239
29	C	0,224216	0,224962	0,000746
30	O	-0,253441	-0,260095	-0,006654
31	O	-0,158044	-0,167111	-0,009067
32	H	0,134908	0,132799	-0,002109
33	C	-0,044795	-0,048776	-0,003981
34	H	0,040255	0,032012	-0,008243
35	H	0,041286	0,028879	-0,012407
36	C	0,013583	0,014428	0,000845

37	C	0.083821	-0.023122	-0.106943	37	C	-0,052692	-0,054902	-0,00221
38	C	0.160159	0.078461	-0.081698	38	H	0,05673	0,045843	-0,010887
39	C	0.00259	-0.045797	-0.048387	39	H	0,055943	0,04968	-0,006263
40	C	-0.004029	-0.048723	-0.044694	40	C	0,223113	0,222487	-0,000626
41	C	0.023546	-0.052691	-0.076237	41	O	-0,231536	-0,234359	-0,002823
42	C	0.008944	-0.061405	-0.070349	42	O	-0,154585	-0,172886	-0,018301
43	H	0.0665	0.034615	-0.031885	43	H	0,159889	0,154804	-0,005085
44	H	0.07122	0.037631	-0.033589	44	H	0,020888	0,016265	-0,004623
45	H	0.088389	0.050606	-0.037783	45	C	0,228438	0,220657	-0,007781
46	H	0.074475	0.040776	-0.033699	46	O	-0,223849	-0,246787	-0,022938
47	H	0.030148	0.026754	-0.003394	47	O	-0,146941	-0,167127	-0,020186
48	C	-0.082075	-0.086711	-0.004636	48	H	0,207347	0,19009	-0,017257
49	H	0.042368	0.034006	-0.008362	49	H	0,079346	0,041565	-0,037781
50	H	0.036194	0.030586	-0.005608	50	C	-0,071149	-0,078002	-0,006853
51	H	0.039905	0.036655	-0.00325	51	H	0,042058	0,034719	-0,007339
52	H	0.055106	0.046181	-0.008925	52	H	0,045931	0,037107	-0,008824
53	C	-0.079621	-0.081512	-0.001891	53	H	0,049373	0,0394	-0,009973
54	H	0.027855	0.034725	0.00687	54	C	0,229074	0,224413	-0,004661
55	H	0.053667	0.0441	-0.009567	55	O	-0,206205	-0,230287	-0,024082
56	H	0.04213	0.037514	-0.004616	56	O	-0,073778	-0,103776	-0,029998
57	H	0.037189	0.032782	-0.004407	57	C	0,115177	0,070566	-0,044611
58	C	0.206839	0.200312	-0.006527	58	C	-0,017158	-0,108987	-0,091829
59	O	-0.257627	-0.276738	-0.019111	59	H	0,070119	0,040987	-0,029132
60	O	-0.178298	-0.19885	-0.020552	60	H	0,075303	0,04643	-0,028873
61	H	0.123391	0.118706	-0.004685	61	C	0,031984	0,025415	-0,006569
62	O	-0.062548	-0.198302	-0.135754	62	H	0,033011	0,028536	-0,004475
63	H	0.152785	0.121331	-0.031454	63	H	0,061037	0,047486	-0,013551
64	C	0.061858	0.066956	0.005098	64	O	-0,100449	-0,105726	-0,005277
65	C	-0.091506	-0.106315	-0.014809	65	C	0,2231	0,221896	-0,001204
66	H	0.050404	0.038658	-0.011746	66	O	-0,228165	-0,224916	0,003249
67	H	0.052397	0.04607	-0.006327	67	C	-0,008723	-0,007195	0,001528
68	C	0.031352	0.030744	-0.000608	68	C	-0,042627	-0,058189	-0,015562
69	H	0.062878	0.05333	-0.009548	69	H	0,060473	0,048616	-0,011857
70	H	0.029822	0.034783	0.004961	70	H	0,048937	0,046026	-0,002911
71	O	-0.091784	-0.102178	-0.010394	71	C	-0,079795	-0,082662	-0,002867
72	C	0.229995	0.224393	-0.005602	72	H	0,047763	0,039111	-0,008652
73	C	-0.009519	-0.009291	0.000228	73	H	0,043949	0,04238	-0,001569
74	C	-0.033891	-0.046424	-0.012533	74	H	0,042623	0,041488	-0,001135
75	H	0.062127	0.051407	-0.01072					
76	H	0.05135	0.052333	0.000983					
77	C	-0.080623	-0.084138	-0.003515					
78	H	0.048996	0.040823	-0.008173					
79	H	0.044458	0.040436	-0.004022					
80	H	0.041355	0.039197	-0.002158					
81	O	-0.251123	-0.228084	0.023039					
82	H	0.198126	0.19129	-0.006836					

AH-2-GM2					EU-2 GM3				
#	atom	qN-1	qN	(f^-)	#	atom	qN-1	qN	(f^-)
1	C	0,198874	0,167997	-0,030877	1	H	0.033429	0.031896	-0.001533
2	O	-0,143918	-0,280832	-0,136914	2	C	-0.079344	-0.084587	-0.005243
3	N	0,037025	-0,093799	-0,130824	3	H	0.04647	0.034692	-0.011778
4	H	0,117478	0,09082	-0,026658	4	H	0.031181	0.025682	-0.005499
5	C	0,0501	0,028565	-0,021535	5	C	-0.039455	-0.044293	-0.004838
6	H	0,061178	0,044042	-0,017136	6	H	0.030069	0.02224	-0.007829
7	C	-0,033274	-0,052613	-0,019339	7	H	0.03998	0.035476	-0.004504
8	H	0,065363	0,04368	-0,021683	8	C	-0.026164	-0.024703	0.001461
9	H	0,04336	0,028488	-0,014872	9	H	0.031213	0.03644	0.005227
10	C	0,000142	0,00686	0,006718	10	C	0.207019	0.211757	0.004738
11	N	-0,139035	-0,13775	0,001285	11	O	-0.263133	-0.254841	0.008292
12	C	0,080454	0,06257	-0,017884	12	O	-0.152313	-0.155214	-0.002901
13	H	0,081455	0,068863	-0,012592	13	H	0.13252	0.127813	-0.004707

14	N	-0,020793	-0,035219	-0,014426
15	H	0,172307	0,158908	-0,013399
16	C	-0,003802	-0,020953	-0,017151
17	H	0,065929	0,055465	-0,010464
18	C	-0,021746	-0,023747	-0,002001
19	C	-0,040522	-0,042828	-0,002306
20	H	0,028737	0,025966	-0,002771
21	H	0,049985	0,035921	-0,014064
22	C	-0,021824	-0,019933	0,001891
23	C	-0,039858	-0,043884	-0,004026
24	H	0,036535	0,029769	-0,006766
25	H	0,040566	0,035073	-0,005493
26	C	-0,080811	-0,08582	-0,005009
27	H	0,037755	0,035072	-0,002683
28	H	0,047474	0,036119	-0,011355
29	H	0,030137	0,026367	-0,00377
30	H	0,039114	0,04167	0,002556
31	C	0,216848	0,219694	0,002846
32	O	-0,258347	-0,256627	0,00172
33	O	-0,160953	-0,169596	-0,008643
34	H	0,185973	0,178706	-0,007267
35	C	0,017125	0,017731	0,000606
36	C	0,223453	0,222844	-0,000609
37	O	-0,211887	-0,238188	-0,026301
38	O	-0,082579	-0,086184	-0,003605
39	C	-0,079015	-0,084888	-0,005873
40	H	0,049061	0,038044	-0,011017
41	H	0,036662	0,03402	-0,002642
42	H	0,041155	0,031661	-0,009494
43	H	0,043874	0,033556	-0,010318
44	C	-0,054837	-0,056851	-0,002014
45	H	0,044981	0,031652	-0,013329
46	H	0,010944	0,015616	0,004672
47	C	-0,043807	-0,049132	-0,005325
48	H	0,051394	0,041914	-0,00948
49	H	0,058197	0,04848	-0,009717
50	C	0,215253	0,212636	-0,002617
51	O	-0,27134	-0,272408	-0,001068
52	O	-0,172428	-0,181997	-0,009569
53	H	0,162527	0,159431	-0,003096
54	C	0,236941	0,210344	-0,026597
55	O	-0,182312	-0,256501	-0,074189
56	O	-0,149183	-0,183591	-0,034408
57	H	0,113553	0,10689	-0,006663
58	C	0,087029	0,070773	-0,016256
59	C	-0,033566	-0,089421	-0,055855
60	H	0,06346	0,044548	-0,018912
61	H	0,067705	0,046641	-0,021064
62	C	0,03174	0,03058	-0,00116
63	H	0,059639	0,04713	-0,012509
64	H	0,035015	0,03542	0,000405
65	O	-0,117312	-0,10679	0,010522
66	C	0,213433	0,211891	-0,001542
67	O	-0,237421	-0,260766	-0,023345
68	C	-0,00657	-0,012477	-0,005907
69	C	-0,072362	-0,067814	0,004548
70	H	0,023652	0,033636	0,009984
71	H	0,046989	0,037665	-0,009324
72	C	-0,079067	-0,085627	-0,00656
73	H	0,040109	0,03336	-0,006749
74	H	0,056336	0,046111	-0,010225
75	H	0,041007	0,032491	-0,008516
76	C	0,198874	0,167997	-0,030877

14	C	-0.051941	-0.05301	-0.001069
15	H	0.03782	0.029807	-0.008013
16	H	0.030098	0.01841	-0.011688
17	C	0.059694	-0.015455	-0.075149
18	C	0.151455	0.065593	-0.085862
19	C	-0.034812	-0.06785	-0.033038
20	C	0.028069	-0.062509	-0.090578
21	C	-0.027799	-0.070804	-0.043005
22	C	0.139135	0.067708	-0.071427
23	H	0.058338	0.032953	-0.025385
24	H	0.07532	0.033672	-0.041648
25	H	0.072558	0.038516	-0.034042
26	O	-0.098089	-0.190132	-0.092043
27	H	0.212623	0.173552	-0.039071
28	C	-0.044079	-0.047594	-0.003515
29	H	0.035781	0.02732	-0.008461
30	C	0.014951	0.014741	-0.00021
31	C	0.214028	0.211239	-0.002789
32	O	-0.158112	-0.167351	-0.009239
33	H	0.174329	0.167876	-0.006453
34	O	-0.24728	-0.232975	0.014305
35	C	-0.049374	-0.051379	-0.002005
36	H	0.053805	0.045528	-0.008277
37	H	0.051958	0.051199	-0.000759
38	C	0.215837	0.215238	-0.000599
39	O	-0.233351	-0.249029	-0.015678
40	O	-0.100104	-0.093388	0.006716
41	C	-0.072779	-0.077778	-0.004999
42	H	0.047967	0.044214	-0.003753
43	H	0.042325	0.033908	-0.008417
44	H	0.047833	0.041756	-0.006077
45	O	-0.045872	-0.133345	-0.087473
46	C	0.024478	0.001945	-0.022533
47	H	0.043652	0.025466	-0.018186
48	H	0.072531	0.046704	-0.025827
49	H	0.054006	0.029307	-0.024699
50	H	0.037081	0.032958	-0.004123
51	C	-0.036682	-0.047559	-0.010877
52	H	0.055442	0.032821	-0.022621
53	H	0.042688	0.027224	-0.015464
54	C	-0.0169	-0.020948	-0.004048
55	H	0.011993	0.011798	-0.000195
56	C	0.068971	0.066367	-0.002604
57	C	-0.066705	-0.060611	0.006094
58	H	0.064407	0.061823	-0.002584
59	H	0.045502	0.04948	0.003978
60	C	0.032449	0.0292	-0.003249
61	H	0.055007	0.049855	-0.005152
62	H	0.051936	0.044841	-0.007095
63	O	-0.126422	-0.129187	-0.002765
64	C	0.207284	0.208002	0.000718
65	C	-0.004168	-0.001194	0.002974
66	C	-0.065338	-0.072871	-0.007533
67	H	0.048922	0.043339	-0.005583
68	H	0.039447	0.035187	-0.00426
69	C	-0.085648	-0.085768	-0.00012
70	H	0.040036	0.03551	-0.004526
71	H	0.031149	0.041685	0.010536
72	H	0.043103	0.037301	-0.005802
73	O	-0.260316	-0.255971	0.004345

MBA-1- GM2					MH-1- GM3				
#	atom	qN-1	qN	(f^{-})	#	atom	qN-1	qN	(f^{-})
1	H	0,052921	0,036727	-0,016194	1	C	0,18627	0,183882	-0,002388
2	C	-0,077508	-0,088412	-0,010904	2	O	-0,234098	-0,233428	0,00067
3	H	0,037614	0,028684	-0,00893	3	N	-0,072536	-0,077668	-0,005132
4	H	0,032575	0,02589	-0,006685	4	H	0,107678	0,104318	-0,00336
5	C	-0,035602	-0,043492	-0,00789	5	C	0,034813	0,027719	-0,007094
6	H	0,039131	0,030891	-0,00824	6	H	0,044083	0,040491	-0,003592
7	H	0,039608	0,034231	-0,005377	7	C	-0,039818	-0,04855	-0,008732
8	C	0,018743	0,00995	-0,008793	8	H	0,059973	0,045134	-0,014839
9	C	-0,080544	-0,085308	-0,004764	9	H	0,060176	0,049287	-0,010889
10	H	0,049944	0,034373	-0,015571	10	C	0,050021	0,003889	-0,046132
11	H	0,043383	0,036165	-0,007218	11	N	-0,159946	-0,18048	-0,020534
12	H	0,030903	0,029815	-0,001088	12	C	0,102237	0,049693	-0,052544
13	C	0,175507	0,176536	0,001029	13	H	0,088607	0,065271	-0,023336
14	O	-0,267155	-0,267065	9E-05	14	N	-0,021287	-0,044768	-0,023481
15	N	-0,062252	-0,076505	-0,014253	15	H	0,176786	0,157717	-0,019069
16	H	0,121238	0,11395	-0,007288	16	C	0,038401	-0,030952	-0,069353
17	C	0,015244	0,0108	-0,004444	17	H	0,08876	0,062104	-0,026656
18	H	0,055168	0,043571	-0,011597	18	C	0,011913	0,012513	0,0006
19	H	0,043537	0,043938	0,000401	19	C	-0,044266	-0,043741	0,000525
20	C	-0,046323	-0,051762	-0,005439	20	H	0,027847	0,031792	0,003945
21	H	0,062443	0,052733	-0,00971	21	H	0,018459	0,020802	0,002343
22	H	0,052305	0,044011	-0,008294	22	C	-0,05378	-0,055699	-0,001919
23	C	0,212971	0,21177	-0,001201	23	H	0,031955	0,028929	-0,003026
24	O	-0,283803	-0,284687	-0,000884	24	H	0,019425	0,024721	0,005296
25	O	-0,184926	-0,187325	-0,002399	25	C	-0,023206	-0,024385	-0,001179
26	H	0,123943	0,124167	0,000224	26	C	-0,039108	-0,041875	-0,002767
27	C	-0,031801	-0,045629	-0,013828	27	H	0,043622	0,036751	-0,006871
28	H	0,046801	0,031573	-0,015228	28	H	0,035326	0,029823	-0,005503
29	H	0,041125	0,032211	-0,008914	29	C	0,01424	0,013416	-0,000824
30	C	0,01976	-0,022903	-0,042663	30	C	0,222624	0,218095	-0,004529
31	C	-0,051544	-0,055072	-0,003528	31	O	-0,252497	-0,247226	0,005271
32	H	0,041919	0,021583	-0,020336	32	O	-0,134159	-0,148051	-0,013892
33	H	0,026727	0,023701	-0,003026	33	H	0,200181	0,194574	-0,005607
34	C	0,014707	0,017611	0,002904	34	C	-0,086164	-0,090577	-0,004413
35	C	0,23103	0,232651	0,001621	35	H	0,044112	0,037279	-0,006833
36	O	-0,199977	-0,221051	-0,021074	36	H	0,035119	0,028458	-0,006661
37	O	-0,098088	-0,089085	0,009003	37	H	0,035774	0,031987	-0,003787
38	C	-0,086155	-0,088114	-0,001959	38	C	-0,054127	-0,054928	-0,000801
39	H	0,054548	0,03277	-0,021778	39	H	0,050578	0,044567	-0,006011
40	H	0,035747	0,038362	0,002615	40	H	0,034204	0,039679	0,005475
41	H	0,032089	0,037176	0,005087	41	C	0,225675	0,222916	-0,002759
42	C	-0,054545	-0,057222	-0,002677	42	O	-0,214547	-0,228958	-0,014411
43	H	0,05636	0,047618	-0,008742	43	O	-0,108443	-0,120709	-0,012266
44	H	0,029128	0,031157	0,002029	44	H	0,04653	0,040374	-0,006156
45	C	0,232688	0,229624	-0,003064	45	C	0,203901	0,202558	-0,001343
46	O	-0,216145	-0,228772	-0,012627	46	O	-0,262949	-0,282007	-0,019058
47	O	-0,159023	-0,17411	-0,015087	47	O	-0,17444	-0,175406	-0,000966
48	H	0,143225	0,139302	-0,003923	48	H	0,120238	0,123779	0,003541
49	H	0,058616	0,027016	-0,0316	49	C	-0,080005	-0,082452	-0,002447
50	C	0,300101	0,209227	-0,090874	50	H	0,041164	0,036552	-0,004612
51	O	0,059582	-0,266526	-0,326108	51	H	0,034745	0,031721	-0,003024
52	O	-0,092371	-0,190664	-0,098293	52	H	0,034941	0,029809	-0,005132
53	H	0,159753	0,128048	-0,031705	53	C	0,21634	0,207811	-0,008529
54	C	0,067655	0,070503	0,002848	54	O	-0,238397	-0,267148	-0,028751
55	C	-0,062126	-0,078067	-0,015941	55	O	-0,167877	-0,182307	-0,01443
56	H	0,067181	0,054642	-0,012539	56	H	0,130841	0,129534	-0,001307
57	H	0,06142	0,057752	-0,003668	57	C	-0,081996	-0,085598	-0,003602
58	C	0,035453	0,031511	-0,003942	58	H	0,040695	0,035461	-0,005234
59	H	0,065959	0,056168	-0,009791	59	H	0,032571	0,031208	-0,001363

60	H	0,046711	0,043012	-0,003699
61	O	-0,114326	-0,119036	-0,00471
62	C	0,213185	0,213555	0,00037
63	O	-0,249346	-0,251066	-0,00172
64	C	-0,006013	-0,007051	-0,001038
65	C	-0,045922	-0,050966	-0,005044
66	H	0,056188	0,051062	-0,005126
67	H	0,052512	0,049517	-0,002995
68	C	-0,079553	-0,082065	-0,002512
69	H	0,055271	0,043206	-0,012065
70	H	0,02879	0,033109	0,004319
71	H	0,043521	0,039943	-0,003578

60	H	0,044528	0,037911	-0,006617
61	C	0,091949	0,071599	-0,02035
62	C	-0,046756	-0,100513	-0,053757
63	H	0,06678	0,048225	-0,018555
64	H	0,068306	0,04855	-0,019756
65	C	0,036285	0,02646	-0,009825
66	H	0,062362	0,048866	-0,013496
67	H	0,053833	0,041791	-0,012042
68	O	-0,116388	-0,12346	-0,007072
69	C	0,218518	0,211501	-0,007017
70	C	0,064231	0,001955	-0,062276
71	C	0,009267	-0,076236	-0,085503
72	H	0,056288	0,034059	-0,022229
73	H	0,060048	0,035341	-0,024707
74	C	-0,067594	-0,087382	-0,019788
75	H	0,06547	0,039119	-0,026351
76	H	0,057861	0,034863	-0,022998
77	H	0,045556	0,033695	-0,011861
78	O	-0,218009	-0,254322	-0,036313

**NMP-1- GM2**				
#	atom	qN-1	qN	(f^{-})
1	C	-0,084326	-0,092437	-0,008111
2	H	0,036433	0,030017	-0,006416
3	H	0,03113	0,023096	-0,008034
4	C	0,013458	0,013966	0,000508
5	C	-0,045534	-0,048593	-0,003059
6	H	0,04988	0,030426	-0,019454
7	H	0,027922	0,025143	-0,002779
8	C	0,205369	0,170159	-0,03521
9	O	-0,144501	-0,266636	-0,122135
10	N	0,1057	-0,034517	-0,140217
11	C	0,046933	0,0185	-0,028433
12	C	-0,035595	-0,052786	-0,017191
13	C	-0,033153	-0,046561	-0,013408
14	C	0,029254	0,001419	-0,027835
15	H	0,052222	0,031802	-0,02042
16	H	0,064427	0,040961	-0,023466
17	H	0,048534	0,03401	-0,014524
18	H	0,067792	0,040698	-0,027094
19	H	0,070216	0,035035	-0,035181
20	H	0,069621	0,029968	-0,039653
21	C	-0,040815	-0,043894	-0,003079
22	H	0,050677	0,033861	-0,016816
23	H	0,030288	0,027262	-0,003026
24	C	-0,031957	-0,03011	0,001847
25	C	0,204963	0,210453	0,00549
26	O	-0,248368	-0,258478	-0,01011
27	O	-0,164415	-0,162987	0,001428
28	H	0,153338	0,149143	-0,004195
29	H	0,024687	0,024095	-0,000592
30	C	-0,038903	-0,042228	-0,003325
31	H	0,033879	0,027674	-0,006205
32	H	0,039956	0,03558	-0,004376
33	C	0,014763	0,014485	-0,000278
34	C	-0,052697	-0,051991	0,000706
35	H	0,05434	0,051987	-0,002353
36	H	0,037201	0,042787	0,005586
37	C	0,224653	0,223429	-0,001224
38	O	-0,258268	-0,26555	-0,007282
39	O	-0,16992	-0,173878	-0,003958
40	H	0,070502	0,035754	-0,034748

**NVC-1- GM2**				
#	atom	qN-1	qN	(f^{-})
1	C	0,044426	0,029616	-0,01481
2	N	0,138591	-0,044366	-0,182957
3	C	0,026796	-0,000445	-0,027241
4	H	0,068279	0,031418	-0,036861
5	H	0,059662	0,033825	-0,025837
6	C	-0,027672	-0,053476	-0,025804
7	H	0,041086	0,027086	-0,014
8	H	0,065726	0,032772	-0,032954
9	C	-0,040507	-0,049588	-0,009081
10	H	0,042975	0,028279	-0,014696
11	H	0,055065	0,030801	-0,024264
12	C	-0,038545	-0,04618	-0,007635
13	H	0,034357	0,027681	-0,006676
14	H	0,057852	0,033397	-0,024455
15	C	-0,05068	-0,06051	-0,00983
16	H	0,047071	0,031314	-0,015757
17	H	0,055721	0,034408	-0,021313
18	C	0,209109	0,169617	-0,039492
19	O	-0,132798	-0,292248	-0,15945
20	C	-0,026886	-0,04486	-0,017974
21	H	0,046106	0,030663	-0,015443
22	H	0,058361	0,03332	-0,025041
23	C	-0,022963	-0,021653	0,00131
24	H	0,028774	0,033841	0,005067
25	C	0,219417	0,216897	-0,00252
26	O	-0,249809	-0,275706	-0,025897
27	O	-0,172068	-0,175073	-0,003005
28	H	0,196621	0,183667	-0,012954
29	C	-0,049835	-0,049405	0,00043
30	H	0,045047	0,0336	-0,011447
31	H	0,019956	0,026774	0,006818
32	C	0,016814	0,017842	0,001028
33	C	-0,092312	-0,096357	-0,004045
34	H	0,027211	0,027552	0,000341
35	H	0,040093	0,032932	-0,007161
36	H	0,038823	0,029631	-0,009192
37	C	0,217757	0,219508	0,001751
38	O	-0,247645	-0,231853	0,015792
39	O	-0,088427	-0,095919	-0,007492
40	C	-0,044279	-0,04742	-0,003141

41	C	0,244891	0,203726	-0,041165
42	O	-0,15966	-0,274023	-0,114363
43	O	-0,141212	-0,197444	-0,056232
44	H	0,142992	0,127369	-0,015623
45	C	-0,081975	-0,086641	-0,004666
46	H	0,044254	0,033204	-0,01105
47	H	0,017853	0,027427	0,009574
48	H	0,051299	0,034231	-0,017068
49	H	0,042848	0,023839	-0,019009
50	C	0,227568	0,226727	-0,000841
51	O	-0,241829	-0,233151	0,008678
52	O	-0,089544	-0,094809	-0,005265
53	C	-0,086353	-0,090677	-0,004324
54	H	0,035435	0,03159	-0,003845
55	H	0,038455	0,03116	-0,007295
56	H	0,03721	0,032799	-0,004411
57	C	0,065186	0,068143	0,002957
58	C	-0,065	-0,075575	-0,010575
59	H	0,059402	0,052868	-0,006534
60	H	0,062446	0,05757	-0,004876
61	C	0,033978	0,034092	0,000114
62	H	0,060956	0,056538	-0,004418
63	H	0,037124	0,044392	0,007268
64	O	-0,110981	-0,116741	-0,00576
65	C	0,218172	0,216962	-0,00121
66	C	-0,012041	-0,008547	0,003494
67	C	-0,041521	-0,055238	-0,013717
68	H	0,057567	0,049903	-0,007664
69	H	0,052619	0,047029	-0,00559
70	C	-0,082429	-0,082359	7E-05
71	H	0,034289	0,039857	0,005568
72	H	0,036133	0,038049	0,001916
73	H	0,046713	0,040711	-0,006002
74	O	-0,238822	-0,221999	0,016823
75	H	0,194201	0,187835	-0,006366

41	H	0,059047	0,051499	-0,007548
42	H	0,042168	0,042193	2,5E-05
43	C	0,215415	0,21431	-0,001105
44	O	-0,247232	-0,259825	-0,012593
45	O	-0,178267	-0,179036	-0,000769
46	C	-0,030718	-0,050432	-0,019714
47	H	0,039371	0,025135	-0,014236
48	H	0,061269	0,032681	-0,028588
49	C	-0,076847	-0,086939	-0,010092
50	H	0,048134	0,034137	-0,013997
51	H	0,023814	0,023926	0,000112
52	H	0,056307	0,03137	-0,024937
53	H	0,051785	0,035973	-0,015812
54	C	0,073483	0,066874	-0,006609
55	C	-0,079382	-0,075535	0,003847
56	H	0,059183	0,050311	-0,008872
57	H	0,041229	0,04927	0,008041
58	C	0,033469	0,031359	-0,00211
59	H	0,058126	0,052077	-0,006049
60	H	0,038849	0,03771	-0,001139
61	O	-0,111216	-0,111412	-0,000196
62	C	0,221741	0,220675	-0,001066
63	O	-0,24388	-0,248496	-0,004616
64	C	-0,005303	-0,004553	0,00075
65	C	-0,051844	-0,057549	-0,005705
66	H	0,054163	0,047929	-0,006234
67	H	0,044347	0,044918	0,000571
68	C	-0,083095	-0,085344	-0,002249
69	H	0,042969	0,037665	-0,005304
70	H	0,037412	0,037756	0,000344
71	H	0,041409	0,03753	-0,003879
72	H	0,146785	0,142453	-0,004332

**NVP-1- GM2**				
#	atom	qN-1	qN	(f^{-})
1	C	0,208154	0,166679	-0,041475
2	C	-0,036557	-0,055587	-0,01903
3	C	-0,03512	-0,050516	-0,015396
4	C	0,033986	0,001151	-0,032835
5	H	0,068157	0,044167	-0,02399
6	H	0,068835	0,041887	-0,026948
7	H	0,043483	0,025981	-0,017502
8	H	0,066048	0,037763	-0,028285
9	H	0,074794	0,032546	-0,042248
10	H	0,083278	0,029153	-0,054125
11	N	0,125007	-0,045363	-0,17037
12	O	-0,124996	-0,280917	-0,155921
13	C	0,054907	0,038006	-0,016901
14	C	-0,032699	-0,048945	-0,016246
15	H	0,046812	0,035325	-0,011487
16	H	0,052811	0,027605	-0,025206
17	C	-0,03276	-0,032466	0,000294
18	H	0,016872	0,020987	0,004115
19	C	-0,049468	-0,049772	-0,000304
20	H	0,039373	0,029914	-0,009459
21	H	0,015879	0,024269	0,00839
22	C	0,018543	0,018044	-0,000499
23	C	0,214281	0,210327	-0,003954
24	O	-0,164271	-0,174187	-0,009916

**ABA-1- GM2**				
#	atom	qN-1	qN	(f^{-})
1	H	0,050201	0,034388	-0,015813
2	C	-0,08232	-0,088411	-0,006091
3	H	0,026699	0,027912	0,001213
4	H	0,034486	0,02524	-0,009246
5	C	-0,04082	-0,045489	-0,004669
6	H	0,044564	0,02844	-0,016124
7	H	0,037102	0,034604	-0,002498
8	C	-0,016206	-0,021385	-0,005179
9	C	0,197864	0,172155	-0,025709
10	O	-0,154318	-0,273267	-0,118949
11	N	-0,005593	-0,08604	-0,080447
12	H	0,122622	0,095627	-0,026995
13	C	0,01448	0,001973	-0,012507
14	H	0,045537	0,040272	-0,005265
15	H	0,058172	0,03195	-0,026222
16	C	-0,043038	-0,051342	-0,008304
17	H	0,074622	0,05802	-0,016602
18	H	0,042166	0,03995	-0,002216
19	C	0,22922	0,228827	-0,000393
20	O	-0,238487	-0,255828	-0,017341
21	O	-0,160154	-0,163046	-0,002892
22	H	0,195177	0,184089	-0,011088
23	C	-0,041822	-0,045912	-0,00409
24	H	0,032908	0,025573	-0,007335

25	H	0,186126	0,1735	-0,012626	25	H	0,052246	0,036807	-0,015439
26	O	-0,240015	-0,259486	-0,019471	26	C	-0,035994	-0,034123	0,001871
27	C	0,218504	0,219381	0,000877	27	H	0,009769	0,017363	0,007594
28	O	-0,216762	-0,234284	-0,017522	28	C	0,215065	0,21427	-0,000795
29	O	-0,094856	-0,089381	0,005475	29	O	-0,236874	-0,257912	-0,021038
30	C	-0,078763	-0,083197	-0,004434	30	O	-0,171333	-0,169171	0,002162
31	H	0,031026	0,030986	-4E-05	31	H	0,191155	0,181256	-0,009899
32	H	0,044947	0,038297	-0,00665	32	C	-0,052166	-0,053717	-0,001551
33	H	0,048075	0,036765	-0,01131	33	H	0,030434	0,030699	0,000265
34	C	-0,048902	-0,049524	-0,000622	34	H	0,043734	0,031178	-0,012556
35	H	0,034627	0,040337	0,00571	35	C	0,016858	0,016174	-0,000684
36	H	0,056858	0,045104	-0,011754	36	C	0,224834	0,216534	-0,0083
37	C	0,215836	0,21559	-0,000246	37	O	-0,223238	-0,252927	-0,029689
38	O	-0,161661	-0,145089	0,016572	38	O	-0,070455	-0,093687	-0,023232
39	H	0,146095	0,146624	0,000529	39	C	-0,046204	-0,050871	-0,004667
40	O	-0,241133	-0,263989	-0,022856	40	H	0,063549	0,054927	-0,008622
41	H	0,076475	0,031875	-0,0446	41	H	0,054151	0,04457	-0,009581
42	C	-0,044199	-0,049232	-0,005033	42	C	-0,073061	-0,078987	-0,005926
43	H	0,037229	0,028829	-0,0084	43	H	0,040421	0,036757	-0,003664
44	H	0,040853	0,026917	-0,013936	44	H	0,045817	0,0385	-0,007317
45	C	-0,06944	-0,081669	-0,012229	45	H	0,050162	0,038123	-0,012039
46	H	0,043597	0,032117	-0,01148	46	C	0,226619	0,222517	-0,004102
47	H	0,0561	0,035203	-0,020897	47	O	-0,245753	-0,233393	0,01236
48	H	0,043183	0,032763	-0,01042	48	O	-0,159587	-0,174218	-0,014631
49	C	0,07209	0,076053	0,003963	49	H	0,148826	0,144238	-0,004588
50	C	-0,088306	-0,100736	-0,01243	50	H	0,034392	0,034042	-0,00035
51	H	0,047508	0,042676	-0,004832	51	C	0,145071	0,070141	-0,07493
52	H	0,057843	0,047873	-0,00997	52	C	0,034476	-0,086918	-0,121394
53	C	0,033702	0,031347	-0,002355	53	H	0,085608	0,047536	-0,038072
54	H	0,056794	0,049032	-0,007762	54	H	0,084296	0,048804	-0,035492
55	H	0,046994	0,043453	-0,003541	55	C	0,035184	0,025257	-0,009927
56	O	-0,117784	-0,113326	0,004458	56	H	0,066709	0,044653	-0,022056
57	C	0,208178	0,205728	-0,00245	57	H	0,045158	0,031979	-0,013179
58	O	-0,240441	-0,262036	-0,021595	58	O	-0,106539	-0,111243	-0,004704
59	C	0,009265	-0,00923	-0,018495	59	C	0,213992	0,211713	-0,002279
60	C	-0,061609	-0,050878	0,010731	60	O	-0,243564	-0,264401	-0,020837
61	H	0,033377	0,038273	0,004896	61	C	-0,0039	-0,011731	-0,007831
62	H	0,040438	0,04468	0,004242	62	C	-0,06083	-0,067882	-0,007052
63	C	-0,07935	-0,085551	-0,006201	63	H	0,024916	0,032616	0,0077
64	H	0,046352	0,038666	-0,007686	64	H	0,055527	0,04167	-0,013857
65	H	0,055909	0,042564	-0,013345	65	C	-0,076672	-0,084543	-0,007871
66	H	0,039811	0,036816	-0,002995	66	H	0,047588	0,037686	-0,009902
					67	H	0,048931	0,04093	-0,008001
					68	H	0,047668	0,036531	-0,011137

**AG-1- GM1**					**MG-1- GM1**				
#	atom	qN-1	qN	(f^{-})	#	atom	qN-1	qN	(f^{-})
1	H	0.038873	0.033745	-0.005128	1	H	0.046874	0.036335	-0.010539
2	C	-0.078059	-0.082626	-0.004567	2	C	-0.079469	-0.085103	-0.005634
3	H	0.041665	0.034137	-0.007528	3	H	0.034976	0.028261	-0.006715
4	H	0.031591	0.028117	-0.003474	4	H	0.031715	0.028559	-0.003156
5	C	-0.049409	-0.050288	-0.000879	5	C	-0.04025	-0.043008	-0.002758
6	H	0.024431	0.024423	-8E-06	6	H	0.035261	0.029113	-0.006148
7	H	0.031934	0.029678	-0.002256	7	H	0.034991	0.034005	-0.000986
8	C	-0.017538	-0.018979	-0.001441	8	C	0.00963	0.010698	0.001068
9	C	0.169693	0.164259	-0.005434	9	C	0.16592	0.16648	0.00056
10	O	-0.293573	-0.275207	0.018366	10	O	-0.267089	-0.259093	0.007996
11	N	-0.080566	-0.087737	-0.007171	11	N	-0.075908	-0.085315	-0.009407
12	H	0.126392	0.11746	-0.008932	12	H	0.13383	0.123718	-0.010112
13	C	-0.00258	-0.002533	4.7E-05	13	C	0.005778	0.003548	-0.00223
14	H	0.025128	0.038897	0.013769	14	H	0.069132	0.056919	-0.012213
15	H	0.064364	0.053812	-0.010552	15	H	0.035508	0.048213	0.012705

16	C	0.216873	0.213234	-0.003639	16	C	0.212602	0.212068	-0.000534
17	O	-0.285424	-0.27668	0.008744	17	O	-0.275852	-0.282984	-0.007132
18	O	-0.164011	-0.18257	-0.018559	18	O	-0.17381	-0.182469	-0.008659
19	H	0.198133	0.187434	-0.010699	19	H	0.197721	0.187676	-0.010045
20	C	-0.040605	-0.042372	-0.001767	20	C	-0.041695	-0.044049	-0.002354
21	H	0.038043	0.040678	0.002635	21	H	0.032101	0.034035	0.001934
22	H	0.036432	0.02777	-0.008662	22	H	0.043671	0.030449	-0.013222
23	C	-0.023253	-0.023821	-0.000568	23	C	-0.024453	-0.027469	-0.003016
24	H	0.034777	0.032003	-0.002774	24	H	0.03588	0.024204	-0.011676
25	C	0.215202	0.211883	-0.003319	25	C	0.227204	0.21198	-0.015224
26	O	-0.244499	-0.220666	0.023833	26	O	-0.202997	-0.242233	-0.039236
27	O	-0.091511	-0.096706	-0.005195	27	O	-0.021869	-0.086167	-0.064298
28	C	-0.051978	-0.052677	-0.000699	28	C	-0.042891	-0.043593	-0.000702
29	H	0.03139	0.027988	-0.003402	29	H	0.046688	0.034509	-0.012179
30	H	0.033491	0.033629	0.000138	30	H	0.04107	0.043257	0.002187
31	C	0.014515	0.014208	-0.000307	31	C	0.014267	0.015091	0.000824
32	C	0.228504	0.228376	-0.000128	32	C	0.230087	0.229476	-0.000611
33	O	-0.236658	-0.240582	-0.003924	33	O	-0.223977	-0.22579	-0.001813
34	O	-0.143552	-0.145698	-0.002146	34	O	-0.13651	-0.145641	-0.009131
35	H	0.202199	0.198503	-0.003696	35	H	0.205602	0.197569	-0.008033
36	C	-0.053532	-0.05529	-0.001758	36	C	-0.05175	-0.054619	-0.002869
37	H	0.049378	0.043154	-0.006224	37	H	0.054366	0.04769	-0.006676
38	H	0.050738	0.04805	-0.002688	38	H	0.056114	0.04363	-0.012484
39	C	-0.07559	-0.078942	-0.003352	39	C	-0.074921	-0.08128	-0.006359
40	H	0.04426	0.041943	-0.002317	40	H	0.041419	0.033624	-0.007795
41	H	0.044301	0.038162	-0.006139	41	H	0.047388	0.042057	-0.005331
42	H	0.038894	0.035276	-0.003618	42	H	0.046562	0.03665	-0.009912
43	C	0.211695	0.20975	-0.001945	43	C	0.203843	0.202575	-0.001268
44	O	-0.271359	-0.261284	0.010075	44	O	-0.267675	-0.270072	-0.002397
45	O	-0.177907	-0.17632	0.001587	45	O	-0.192193	-0.187456	0.004737
46	H	0.138933	0.135881	-0.003052	46	H	0.126849	0.121313	-0.005536
47	H	0.05078	0.044442	-0.006338	47	C	-0.085698	-0.089354	-0.003656
48	C	0.082399	0.086643	0.004244	48	H	0.042368	0.032367	-0.010001
49	C	-0.079367	-0.103717	-0.02435	49	H	0.043094	0.034469	-0.008625
50	H	0.052811	0.037925	-0.014886	50	H	0.028982	0.030014	0.001032
51	H	0.056822	0.047733	-0.009089	51	C	0.221803	0.078172	-0.143631
52	C	0.0407	0.029466	-0.011234	52	C	0.129292	-0.092349	-0.221641
53	H	0.059375	0.045439	-0.013936	53	H	0.097137	0.040241	-0.056896
54	H	0.051595	0.040795	-0.0108	54	H	0.112435	0.047472	-0.064963
55	O	-0.097366	-0.113551	-0.016185	55	C	0.050622	0.032756	-0.017866
56	C	0.225879	0.210774	-0.015105	56	H	0.070409	0.050071	-0.020338
57	O	-0.19268	-0.270115	-0.077435	57	H	0.070249	0.03855	-0.031699
58	C	0.173671	-0.013623	-0.187294	58	O	-0.105439	-0.112771	-0.007332
59	C	0.168534	-0.058936	-0.22747	59	C	0.215962	0.206474	-0.009488
60	H	0.081275	0.027858	-0.053417	60	C	-0.005645	-0.011209	-0.005564
61	H	0.109708	0.043389	-0.066319	61	C	-0.057969	-0.071686	-0.013717
62	C	-0.041002	-0.085368	-0.044366	62	H	0.028335	0.03108	0.002745
63	H	0.093046	0.035419	-0.057627	63	H	0.052084	0.037462	-0.014622
64	H	0.067116	0.034696	-0.03242	64	O	-0.241862	-0.275261	-0.033399
65	H	0.096354	0.039095	-0.057259	65	C	-0.078576	-0.08582	-0.007244
					66	H	0.045297	0.033767	-0.01153
					67	H	0.048244	0.03999	-0.008254
					68	H	0.045213	0.038271	-0.006942

MGA-1- GM2

#	atom	qN-1	qN	(f^{-})	#	atom	qN-1	qN	(f^{-})
1	H	0,043031	0,036047	-0,006984	40	O	-0,196744	-0,194005	0,002739
2	C	-0,076643	-0,080851	-0,004208	41	H	0,118658	0,125351	0,006693
3	H	0,036665	0,033348	-0,003317	42	C	-0,064688	-0,062204	0,002484
4	H	0,040422	0,035583	-0,004839	43	H	0,006248	0,018807	0,012559
5	C	-0,045542	-0,046206	-0,000664	44	H	0,029635	0,029339	-0,000296
6	H	0,024186	0,025322	0,001136	45	C	0,017105	0,017653	0,000548

7	H	0,029918	0,027736	-0,002182	46	C	0,218049	0,219259	0,00121
8	C	0,013295	0,013438	0,000143	47	O	-0,243445	-0,253168	-0,009723
9	C	-0,085129	-0,087882	-0,002753	48	O	-0,102829	-0,099308	0,003521
10	H	0,03496	0,031276	-0,003684	49	C	-0,04287	-0,046891	-0,004021
11	H	0,04556	0,038037	-0,007523	50	H	0,058192	0,049519	-0,008673
12	H	0,030993	0,031275	0,000282	51	H	0,060885	0,052463	-0,008422
13	C	0,189209	0,18527	-0,003939	52	C	-0,075066	-0,079851	-0,004785
14	O	-0,245775	-0,229982	0,015793	53	H	0,046051	0,039428	-0,006623
15	N	-0,072318	-0,0751	-0,002782	54	H	0,038658	0,033799	-0,004859
16	H	0,123474	0,116808	-0,006666	55	H	0,044612	0,038535	-0,006077
17	C	0,017251	0,020755	0,003504	56	C	0,233587	0,226678	-0,006909
18	H	0,019391	0,032679	0,013288	57	O	-0,261951	-0,232225	0,029726
19	C	0,225514	0,225285	-0,000229	58	O	-0,156025	-0,1721	-0,016075
20	O	-0,256149	-0,243988	0,012161	59	C	0,07467	0,079656	0,004986
21	O	-0,142463	-0,159975	-0,017512	60	C	-0,067822	-0,089621	-0,021799
22	H	0,204476	0,195566	-0,00891	61	H	0,061416	0,053231	-0,008185
23	C	-0,036491	-0,040819	-0,004328	62	H	0,055033	0,041807	-0,013226
24	H	0,041602	0,036693	-0,004909	63	C	0,034268	0,024393	-0,009875
25	H	0,048576	0,036536	-0,01204	64	H	0,054576	0,043778	-0,010798
26	C	-0,048422	-0,053643	-0,005221	65	H	0,046906	0,037171	-0,009735
27	H	0,03999	0,034599	-0,005391	66	O	-0,094923	-0,106238	-0,011315
28	H	0,065408	0,052511	-0,012897	67	C	0,217724	0,207716	-0,010008
29	C	0,219566	0,215906	-0,00366	68	O	-0,19182	-0,261603	-0,069783
30	O	-0,244624	-0,262673	-0,018049	69	C	0,170568	-0,007547	-0,178115
31	O	-0,179266	-0,167102	0,012164	70	C	0,153482	-0,07553	-0,229012
32	H	0,137423	0,14078	0,003357	71	H	0,106445	0,04029	-0,066155
33	C	-0,045278	-0,04607	-0,000792	72	H	0,082412	0,026503	-0,055909
34	H	0,041669	0,034804	-0,006865	73	C	-0,0433	-0,085824	-0,042524
35	H	0,022234	0,022158	-7,6E-05	74	H	0,091619	0,037442	-0,054177
36	C	-0,024249	-0,02481	-0,000561	75	H	0,065565	0,034415	-0,03115
37	H	0,041156	0,035047	-0,006109	76	H	0,094158	0,038336	-0,055822
38	C	0,207028	0,206516	-0,000512	77	H	0,1895	0,192081	0,002581
39	O	-0,268892	-0,286138	-0,017246					

Table S3. Gibb free energy of the glass ionomer molecules (E_{GIC}), modified polymer (E_P), metal atoms (E_M) and interaction energy (E_i) in Hartree. Calculated at M06/6-311G(d,p)

Molecule	E_{GIC}	E_P	E_M	E_i
AACA-1- GM3-Ca	-2571.969795		Ca: -677.559624	0.965729
AACA-1- GM3-Zn	-3673.480954	-1895.3759	Zn: -1779.230858	1.125804
AACA-1- GM3-Al	-2136.185391		Al: -242.367728	1.558237
AADH-1- GM3-Ca	-2724.269088			0.964736
AADH-1- GM3-Zn	-3825.743167	-2047.6742		1.161891
AADH-1- GM3-Al	-2288.428901			1.613027
AADOH-3- GM3-Ca	-2799.47796			0.966217
AADOH-3- GM3-Zn	-3900.946782	-2122.884553		1.168629
AADOH-3- GM3-Al	-2363.531028			1.721253
ABA-1- GM2-Ca	-2454.169012			0.962179
ABA-1- GM2-Zn	-3555.668337	-1777.571567		1.134088
ABA-1- GM2-Al	-2018.362941			1.576354
AG-1-GM1-Ca	-2414.874214			0.962179
AG-1-GM1-Zn	-3516.370548	-1738.287167		1.134088
AG-1-GM1-Al	-1979.05695			1.576354
AGA-2- GM2-Ca	-2681.9934			0.944139
AGA-2- GM2-Zn	-3783.488178	-2005.377915		1.120595
AGA-2- GM2-Al	-2246.194971			1.550672
AH-2- GM2-Ca	-2679.056654			0.95241
AH-2- GM2-Zn	-3780.540674	-2002.449440		1.139624
AH-2- GM2-Al	-2243.228269			1.588899
EU-2- GM3-Ca	-2478.250977			0.944463
EU-2- GM3-Zn	-3579.726855	-1801.635816		1.139819
EU-2- GM3-Al	-2042.783131			1.220413
MBA-1- GM2-Ca	-2493.43679			0.963159
MBA-1- GM2-Zn	-3594.939606	-1816.840325		1.131577
MBA-1- GM2-Al	-2057.640636			1.567417
MG-1- GM1-Ca	-2454.149232			0.969037
MG-1- GM1-Zn	-3555.639675	-1777.558645		1.149828
MG-1- GM1-Al	-2018.271977			1.654396
MGA-1- GM2-Ca	-2721.196926			0.987918
MGA-1- GM2-Zn	-3822.715217	-2044.62522		1.140861
MGA-1- GM2-Al	-2285.426815			1.566133
MH-1- GM3-Ca	-2718.313605			0.963901
MH-1- GM3-Zn	-3819.807991	-2041.717882		1.140749
MH-1- GM3-Al	-2282.546208			1.539402
NMP-1- GM2-Ca	-2570.747451			0.979992
NMP-1- GM2-Zn	-3672.252199	-1894.167819		1.146478
NMP-1- GM2-Al	-2134.839503			1.696044
NVC-1- GM2-Ca	-2382.192909			1.001583
NVC-1- GM2-Zn	-3483.71965	-1705.634868		1.146076
NVC-1- GM2-Al	-1946.710745			1.291851
NVP-1- GM2-Ca	-2303.692136			0.967644
NVP-1- GM2-Zn	-3405.194127	-1627.100156		1.136887
NVP-1- GM2-Al	-1868.32228			1.145604

AACA-1-GM3	AADH-1-GM3
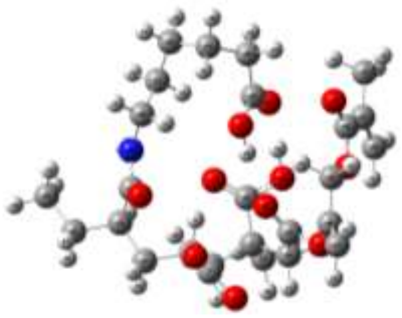	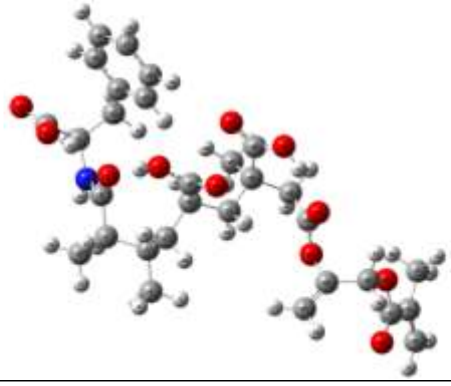
AADOH-3-GM3	AGA-2-GM2
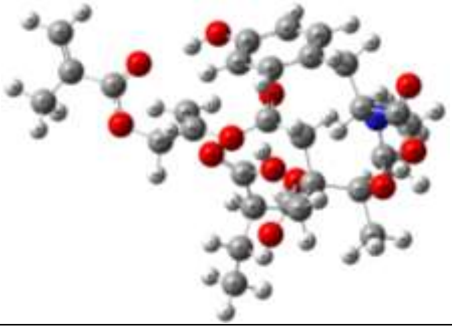	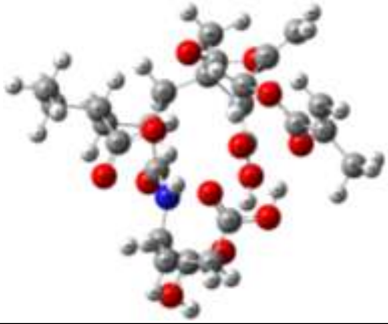
AH-2-GM2	EU-2-GM3
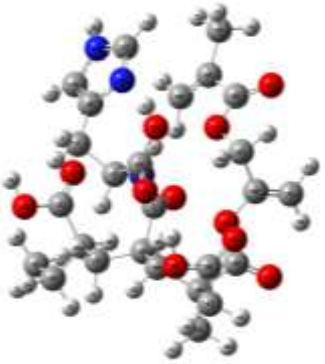	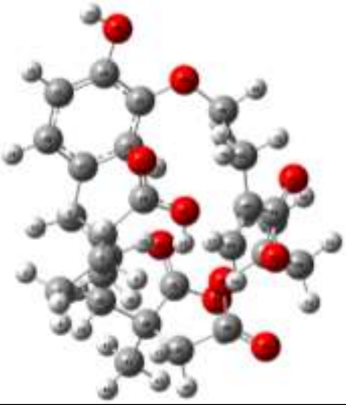
MBA-1-GM2	MH-1-GM3
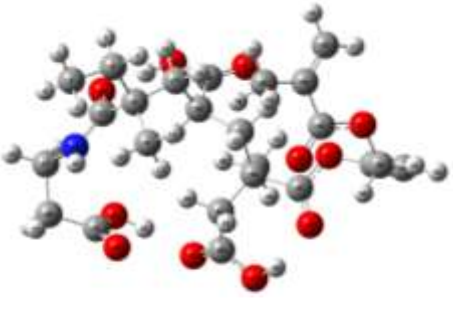	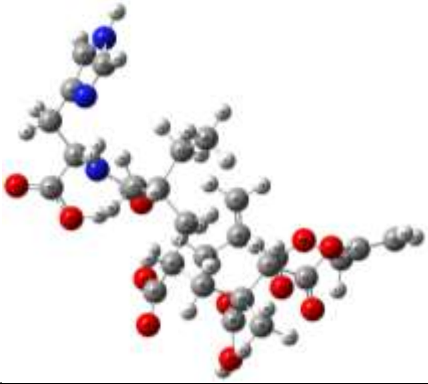
NMP-1-GM2	NVC-1-GM2

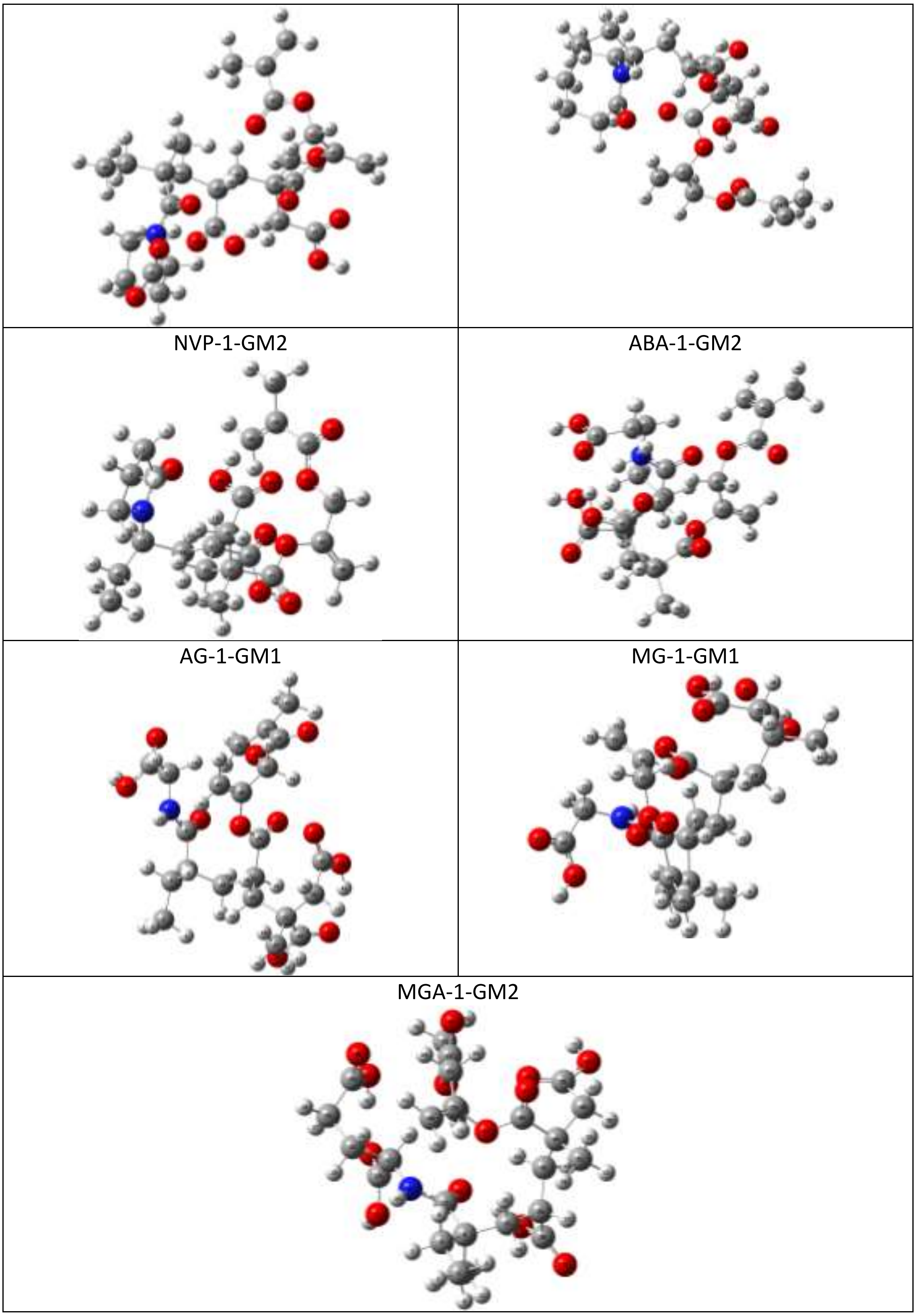

Figure S1. More stable molecules obtained from the addition reaction of glycidyl methacrylate on the polyacids studied. Optimized to M06-2X/6-311G(d,p)
